# Cannabinoids: Role in Neurological Diseases and Psychiatric Disorders

**DOI:** 10.3390/ijms26010152

**Published:** 2024-12-27

**Authors:** Ujendra Kumar

**Affiliations:** Faculty of Pharmaceutical Sciences, The University of British Columbia, Vancouver, BC V6T 1Z3, Canada; ujkumar@mail.ubc.ca

**Keywords:** cannabinoid, endocannabinoid, receptor, neurological diseases, signaling

## Abstract

An impact of legalization and decriminalization of marijuana is the gradual increase in the use of cannabis for recreational purposes, which poses a potential threat to society and healthcare systems worldwide. However, the discovery of receptor subtypes, endogenous endocannabinoids, and enzymes involved in synthesis and degradation, as well as pharmacological characterization of receptors, has led to exploration of the use of cannabis in multiple peripheral and central pathological conditions. The role of cannabis in the modulation of crucial events involving perturbed physiological functions and disease progression, including apoptosis, inflammation, oxidative stress, perturbed mitochondrial function, and the impaired immune system, indicates medicinal values. These events are involved in most neurological diseases and prompt the gradual progression of the disease. At present, several synthetic agonists and antagonists, in addition to more than 70 phytocannabinoids, are available with distinct efficacy as a therapeutic alternative in different pathological conditions. The present review aims to describe the use of cannabis in neurological diseases and psychiatric disorders.

## 1. Introduction

**Figure 1:**
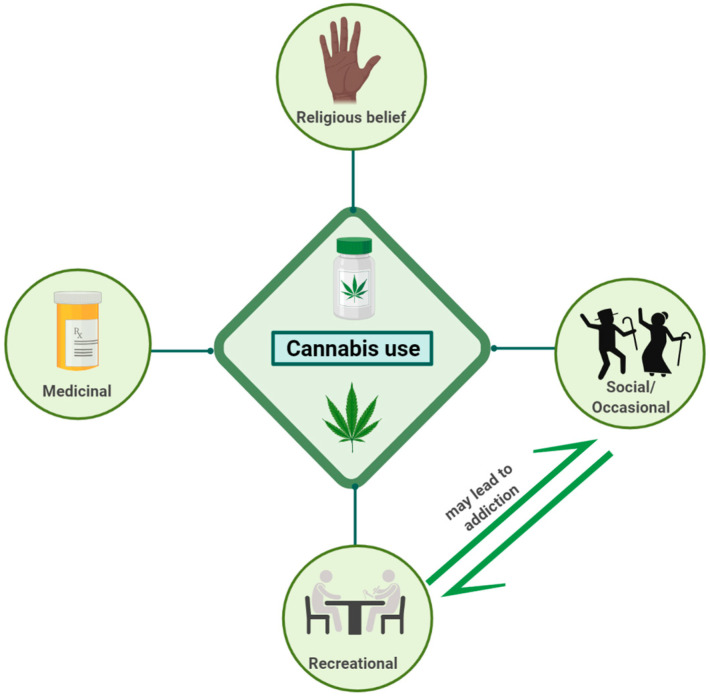
Cannabis use and different beliefs. This figure was created with BioRender.com.

*Cannabis sativa* L. (marijuana), an ancient plant with medicinal values, has been used for medicinal, recreational, and spiritual purposes for a long time worldwide [1,2]. In the 19th century, cannabis was introduced to the western countries, primarily for medicinal uses [3]. Past studies have shown a variable number of chemicals extracted from this hemp plant (*Cannabis sativa* L.) with beneficial, psychoactive, and adverse effects. At present, more than 500 different chemical components have been detected, including more than 70 phytocannabinoids (Phyto-CBs) with some medicinal values; amongst them, Δ^9^-tetrahydrocannabinol (Δ^9^-THC) is the most prominent and best-studied cannabinoid with psychoactive ingredients. Δ^9^-THC, in addition to medicinal values, is also associated with psychoactive effects. Cannabinol was the first cannabinoid extracted as a plant compound, followed by cannabidiol (CBD). In addition, several aromatic terpenes are also key constituent of cannabis. The wide range of therapeutic implications of cannabis includes sensation, memory, antiemetics, analgesic drugs, pain, anticonvulsants, neuromodulation, and lowered intraocular pressure [4,5]. Earlier studies have also demonstrated the appetite stimulatory role of cannabinoids in human immunodeficiency virus (HIV) and cancer patients [6,7,8]. However, the therapeutic use of cannabinoids was hampered due to psychotic effects and addiction as well as tolerance with sustained and prolonged use. Moreover, inflammation, cardiovascular regulation, musculoskeletal disorder, and cancer, which are linked with apoptosis, cell migration, and modulation of immune suppression, has also been reported [5,9,10]. The epidemiological data summarized that cannabis use ranked 3rd as substance abuse, and 192 million people, constituting 3.9% of the world population, use cannabis, and cannabis use is growing at a faster rate than any other illicit drugs [2]. However, it did not take long for cannabis to become illegal after the discovery of the psychoactive effect [3]. Since then, cannabis has been labeled as a narcotic and could not escape such negative connotations [3]. Cannabis, as shown in Figure 1, used either for medicinal purposes or recreationally, is one of the substances of abuse that has been the most controversial drug and is often a center of debate socially, clinically, and politically.

Gaoni and Mechoulam, for the first time, isolated a compound known as Δ^9^-THC [11] with psychoactive activity. This significant observation led to the discovery of a target site for the Δ^9^-THC and two cannabinoid receptors (CBRs), namely Cannabinoid Receptor 1 (CB1R) and Cannabinoid Receptor 2 (CB2R), which were cloned and characterized in 1991 and 1993, respectively [12,13,14]. CBRs, which are abundant in different regions of the brain, were classified as a member of the G protein-coupled receptor family, which couple to the inhibitory G proteins (Gi) and inhibit the enzyme adenylyl cyclase (AC) and suppress the formation of the second messenger cyclic adenosine monophosphate (cAMP) [15]. Further achievements soon after receptor identification led to the discovery of two endogenous cannabinoids known as endocannabinoids (eCB), namely arachidonoyl ethanolamine (AEA), commonly known as anandamide, and 2-arachidonoyl glycerol (2AG), with an ability to work on CBRs [16,17,18,19]. Endogenous ligands, such as 2AG, can play a similar role as Δ^9^-THC [17]. These endogenous ligands for CBR subtypes and enzymes involved in their synthesis and degradation are collectively known as the endocannabinoid system (ECS). The discovery of the eCB sparked the interest in cannabinoid research and, therefore, is a topic of extensive interest in many different disciplines of science globally. Most animal studies exhibiting brain abnormalities observed with use of cannabinoids have not been translated absolutely in humans. In this context, results from earlier studies are most controversial. Such discrepancies are multifactorial, including amount, time, abnormal psychological disturbances, and the duration of drugs used. A conclusive message drawn from past studies suggests that the duration of cannabis used plays a crucial role in brain abnormal morphology and functioning, such as memory and decision making [20]. Taken into consideration, the cloning of CBR genes, the development of selective antibodies, agonists, and antagonists, the discovery of endocannabinoids, and the understanding of transporters and enzymes associated with cannabinoid synthesis and degradation have contributed significantly to the understanding the anatomical distribution of CBRs, and their physiological role, pharmacological properties, and biochemical implication in a receptor-specific manner [13,21,22,23,24,25]. We recently described the role of cannabis and endocannabinoids in the central nervous system (CNS) with some limitations in the clinical use of cannabis [26]. The present review aims to discuss the genetic variation, expression at the level of gene and protein, molecular structure, distributional pattern in different brain regions, associated signaling pathways, and therapeutic implications of cannabinoids in neurological diseases and psychiatric disorders.

## 2. Endocannabinoid System (ECS)

**Figure 2:**
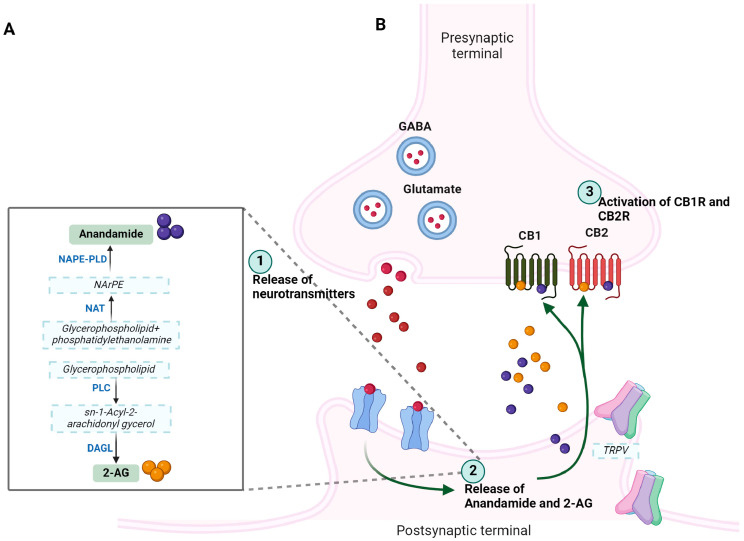
Schematic illustration displaying the synthesis of 2-AG and anandamide (**A**) and endocannabinoid retrograde signaling-mediated synaptic transmission (**B**). (1) Release of neurotransmitters from presynaptic terminals; (2) release of endogenous cannabis on demand from postsynaptic terminal; (3) activation of CB1R and CB2R in the presence of endogenous cannabis anandamide and 2-AG. Endogenous cannabinoids 2-AG and anandamide (AEA) are synthesized—acyl phosphatidylethanolamine. 2-AG and AEA are released from postsynaptic terminals on demand and act on CB1R and CB2R located on presynaptic terminals. Activated CBR subtypes, specifically CB1R, are involved in regulating the release of several neurotransmitters from presynaptic terminal to act on their cognate receptors located at the postsynaptic terminal. 2-AG is removed and processed for degradation, whereas AEA works on other CBR independent target including TRVP. Adopted from Zou. S and Kumar. U, Int. J. Mol. Sci. 2018, 19, 833 [26]. This figure was created with BioRender.com.

The ECS is a complex biological system with a widespread distribution that exerts significant physiological functions not only in normal but also in several pathological conditions, including neurological diseases and psychiatric disorders. Two different cell surface receptors protein, namely CB1R and CB2R, two endogenous ligands (eCBs) to receptors, i.e., AEA and 2-AG, enzymes responsible for synthesis (Diacylglycerol (DAG) lipase and N-acylphosphatidylethanolamine-phospholipase D (NAPE-PLD)) and degradation (Fatty acid amide hydrolase (FAAH) and monoglycerol lipase (MAGL)) of eCB levels, as well as transporters involved in the termination of receptor action are collectively known as ECS. eCBs are hydrophobic lipid-derived compounds primarily referred to as endogenous lipids, which have two common structural motifs, i.e., polyunsaturated fatty acid, also known as arachidonic acid and secondly, ethanolamine and glycerol (Figure 2A,B).

**Figure 3:**
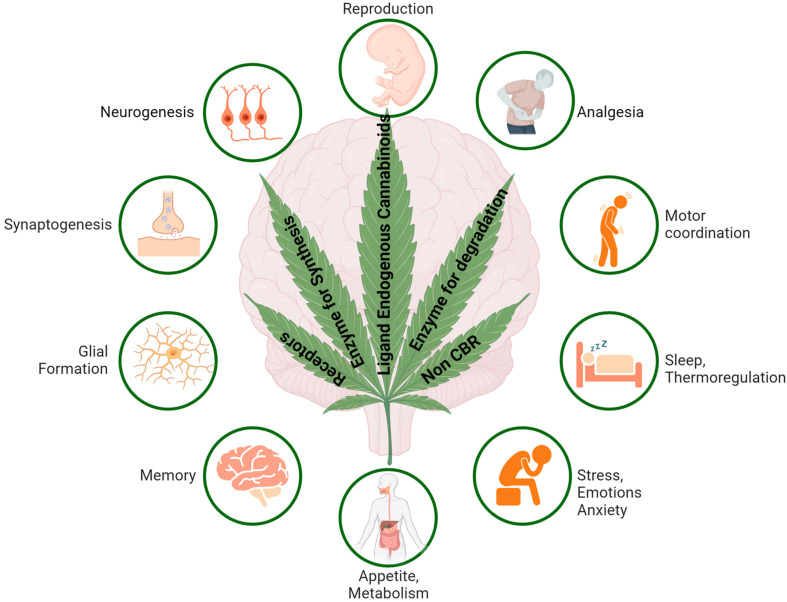
Illustration showing the endocannabinoid system in the brain and associated functions in the human body. This figure was created with BioRender.com.

AEA and 2-AG have been studied the most, which are expressed in neurons, glia, and several other tissues and serve as endogenous ligands to CBRs, which are involved in a different array of functions in CNS, including synaptic plasticity and neuronal development, and are a prominent center of neuromodulation [27,28,29,30]. eCBs are produced on demand, released from the postsynaptic terminal in response to increased intracellular Ca^2+^, bind to and activate CBRs present on presynaptic terminals, and exert negative regulation of neurotransmitters release, a mechanism known as retrograde signaling at central synapses (discussed later). In the brain, the relative concentration of 2-AG is several folds higher than AEA. eCBs are involved in the modulation of several physiological functions in humans through the activation of CB1R and CB2R and are expected to exert a significant role in human diseases. Moreover, studies showing the effect of eCBs in humans are still elusive, despite significant differences in distribution and functions in the target tissue. Moreover, studies have suggested that the eCBs are too complex to have only two receptors [31]. Accordingly, studies support neuronal function and synaptic transmission in response to eCBs independent of CBR subtypes and in a nonretrograde manner via transient receptor potential vanilloid receptor type 1 (TRPV1) and in the presence of CB1R at the postsynaptic site [32]. Begg et al., provided evidence and claimed that the orphan receptor GPR55 is a novel cannabinoid receptor and showed that CP55940, AEA, and 2-AG are possible ligands for the GPR55 receptor [31]. eCBs are not stored in vesicles as normally seen by most neurotransmitters and neuropeptides, but are instead synthesized on demand whenever needed and are involved in enzymatic cleavage in an activity-dependent manner. There are different mechanisms for synthesis and metabolism for both 2AG and AEA. N-acylphosphatidylethanolamine-phospholipase D (NAPE-PLD) and the sn-1-specific diacylglycerol (DAG) lipase (DAGL) are the two enzymes associated with the synthesis of AEA and 2-AG, respectively. NAPE-PLD and DAGL synthesize AEA and 2-AG from the phospholipid membrane [33,34]. On the other hand, when fatty acid amide hydrolase (FAAH), a postsynaptic membrane protein that is associated with degradation of AEA into arachidonic acid and monoacylglyceride lipase (MAGL), whereas presynaptic localization is involved in the degradation of AEA and 2-AG, respectively. Yet controversial, a process of cellular uptake is also involved in the removal of eCBs from the intracellular space. An Additional role of cyclooxygenases, lipooxygenases, and cytochrome P450 has also been proposed in the metabolism of eCBs and the consequent formation of bioactive metabolites predicted a possible mechanism of function in a non-CBR-dependent manner [35]. In addition, several other pathways involved in the modulation of eCBs have also been reported [36]. Such interconnected enzymes associated with the regulation of endogenous expression levels of eCBs might play a crucial role in brain functions in CNS, including firing of neurons or activation of CBR subtypes. Manira and Kyriakatos further stated that distinct independent pathways of synthesis and degradation of AEA and 2-AG might play a critical role in eCBs distribution and function in CNS [37]. In addition to AEA and 2-AG, other endogenous cannabinoids are also produced in the brain, including Docosatetraenoylethanolamide, Homo-γ-linolenyethanolamide, Oleylethanolamide, Palmitylethanolamide, and Stearylethanolamide, which also bind and activate CBR subtypes but are also well known to function independent of CBR subtypes and use TRPV1, GPR55, and PPARα as a possible target. The possible synthesis pathways and related metabolism steps for AEA and 2-AG are shown in Figure 2A,B. A wide variety of physiological functions and effective ability of eCBs in several pathological conditions including pain, inflammation, thermoregulation, memory, behavior, and appetite are attributed to the regulation of multiple neurotransmitter systems in CNS including gamma-aminobutyric acid (GABA), dopamine, opioid, serotonin, and glutamate, as well as neuropeptide by endogenous cannabinoids (Figure 3). The interaction between dopamine and eCB is important in different brain functions, such as motor control, reward, and psychosis in a region-specific manner including the nucleus accumbens, VTA, substantia nigra, striatum, and prefrontal cortex. The modulation of motor behavior involves the basal ganglia with high levels of 2-AG and AEA along with other neurotransmitters including GABA, glutamate, dopamine, and acetylcholine. Moreover, eCB retrograde signals are involved in regulation of GABAergic and glutamatergic neurotransmission in brain regions that are rich with dopamine. Also, in the nucleus accumbens, goal-seeking behavior is associated with eCB signaling and dopamine. eCB interaction with different signaling and neuromodulation systems is not only involved in regulation of glutamate and GABA but can also regulate dopamine, acetylcholine, serotonin, and opioids. These observations support the notion that eCB release in different brain regions might be involved in the multiple physiological functions as stated above.

### 2.1. Arachidonoyl Ethanolamine (AEA): Biosynthesis and Functions

AEA, commonly known as anandamide, was the first endocannabinoid identified, and its synthesis involves multiple pathways. As illustrated in Figure 2, in the process of AEA biosynthesis, two different pathways are proposed. Moreover, AEA is synthesized from phospholipid precursor *N*-arachidonoyl phosphatidylethanolamine (PE), followed by catalyzation in the presence of phospholipase D (PLD)-type activity. AEA is processed for degradation in the presence of the enzyme FAAH and followed by production of arachidonic acid (AA) and ethanolamine [38,39,40,41].

AEA is derived from arachidonic acid and binds to CB1R and relatively to the lesser degree to CB2R in the CNS [42]. There are many speculations about AEA that require further experiments. For instance, it has been demonstrated that AEA can delay the memory of rats and suppress the growth of breast cancer cells [42,43]. Conversely, when bound to CB2 receptors, AEA is involved in functions with the immune system [43]. AEA is present at low levels throughout the body due to fatty acid amide hydrolase enzyme (FAAH), which functions to degrade AEA into arachidonic acid and ethanolamine [43]. The high distributional pattern of CB1R and FAAH in brain regions, i.e., cortex, hippocampus, and cerebellum, display a close relation [44,45]. The pathophysiological significance of AEA first emerged from its inhibitory effect on cell proliferation via apoptosis, specifically in lymphocytes. Although, the mechanism associated with the AEA proapoptotic effect is not well understood despite its in vivo and in vitro proapoptotic effect [46,47]. Previous studies have also proposed the role of glutamate receptor agonist and stimulation of dopamine (DA), specifically dopamine receptor 2 (D2R), in the formation of AEA during neuronal activity in Ca^2+^ and G protein-coupled process-dependent manner, respectively [48,49]. AEA is also known to bind and activate TRPV1. These results indicate the potential therapeutic implication of AEA. During the process of receptor activation, released AEA from the postsynaptic terminal acts on CBR subtypes on the presynaptic terminal and is followed by removal in the presence of the transporter, which is expressed in neuronal cells and astrocytes [50].

### 2.2. 2-Arachidonoylglycerol (2-AG)

2-AG was the second endocannabinoid discovered and served as a full agonist to CBR subtypes [51]. 2-AG binds to CB1R and CB2R with the same affinity but with relatively higher potency than AEA. 2-AG is similar to AEA in a way that both of their derivatives contain arachidonic acid [41]. Unlike AEA, 2-AG is widely expressed and ubiquitous throughout the body, especially in the CNS and plays a critical role in neuronal development and synaptic plasticity. The expression levels of 2-AG in different brain regions including the hippocampus, striatum, and brain stem are several folds higher than AEA. However, its isolation from brain tissues is rather complicated due to its tendency to isomerize into 1-AG [41]. Nevertheless, 2-AG is synthesized from membrane phospholipids through activation of phospholipase Cβ and diacylglycerol lipases that contain arachidonic acid, which is facilitated by the enzyme diacylglycerol lipases [41]. The degradation of 2-AG is a complex process involving multiple enzymes and resulted in either degradation to arachidonic acid (AA) and glycerol or bioactive signaling molecules [52,53]. 2-AG has also been reported to bind GABA-A receptors [54].

In addition to serving as an endogenous ligand to CBR subtypes, studies have also indicated that 2-AG can bind as a potential ligand to many other receptors and broaden their functions indicating physiological effect of 2-AG independent of CBR subtypes. Amongst them, members of the G protein-coupled receptors (GPCRs) family, such as GPR 55 and GPR 119, are the prominent target [55]. Furthermore, 2-AG also works as an agonist to vanilloid type 1 receptor and involves regulating neurotransmitter release. This interaction further supports the link between 2-AG and pain. Furthermore, studies also support the binding of 2-AG to the GABA-A receptor in mice lacking both CBR subtypes supporting the role of 2-AG in sedation and locomotion. These observations emphasize the functional significance of eCB; the mechanism may be different from CBR-mediated regulation of signaling. Taken together, 2-AG can be recognized as a crucial endocannabinoid with broader targets implicating in physiological functions and behavioral changes.

It is also worth mentioning here that the presence of both AEA and 2-AG in the same postsynaptic neurons raises the question regarding the interaction between AEA and 2-AG. It was proposed that both AEA and 2-AG, using different mechanisms, can be recruited at postsynaptic neurons and interact with each other [56,57,58,59]. However, molecular determinants of consequences for such interaction on eCB physiological events are not well understood. Taken together, the regulation of expression levels of eCBs by targeting biological degrading molecules FAAH and MGL might serve as a potential and efficacious therapeutic target in neurological diseases.

## 3. Phytocannabinoids

**Figure 4:**
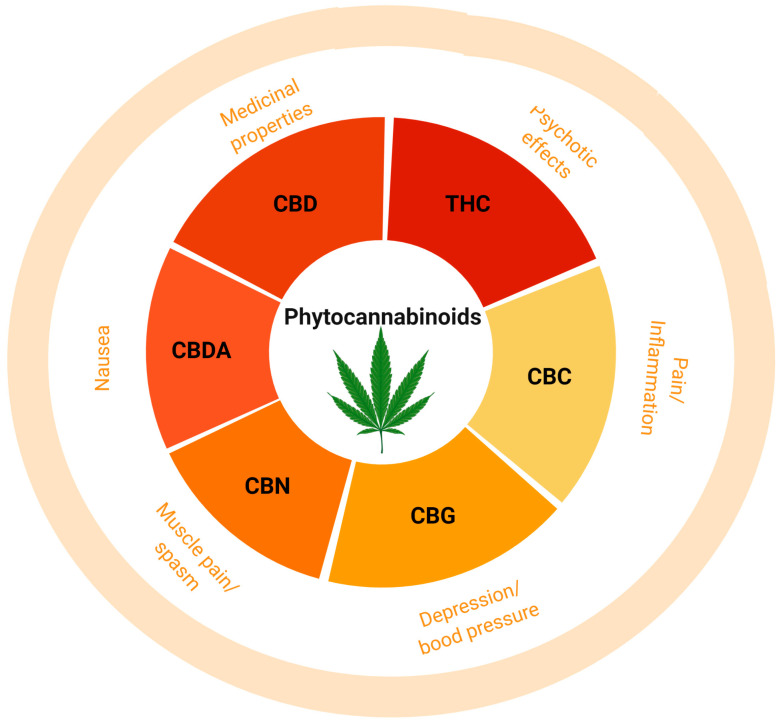
Schematic presentation showing key phytocannabinoids extracted from plant *Cannabis sativa* L. (marijuana) and their associated function in human. This figure was created with BioRender.com.

**Figure 5:**
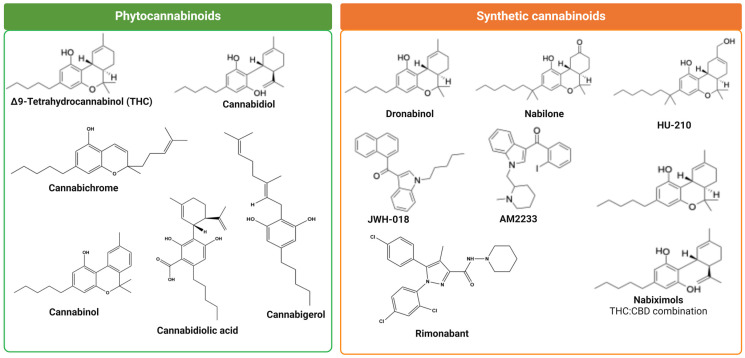
Chemical structure of phytocannabinoids and synthetic cannabinoids.

At present, more than 70 compounds have been isolated from *Cannabis sativa* L. (marijuana) extract, which are commonly known as phytocannabinoids (Phyto-CBs) and, interestingly, each compound has its own distinct pharmacological properties [60,61,62,63,64]. The most studied and well characterized Phyto-CBs includes Δ^9^-THC, cannabidiol (CBD), Cannabigerol (CBG), Cannabinol (CBN), Cannabichromene (CBC), and Cannabidiolic Acid (CBDA), which exist in a form of inactive monocarboxylic acid, the moiety that prevents binding to the target including CBR subtypes [65,66]. Amongst all Phyto-CBs, few have drawn great attention either for their beneficial role in some pathological conditions or also for their disruptive role in normal physiological functions and are discussed here (Figure 4 and Figure 5).

### 3.1. (−)-Trans-Δ^9^-Tetrahydrocannabinol

Δ^9^-THC was first isolated and synthesized in Mechoulam’s laboratory [11] and is one of the cannabinoids from *Cannabis sativa* L. plant extract, which is responsible for most of the psychoactive effects, and that also prompted the synthesis of several synthetic cannabinoids [64,67,68]. Δ^9^-THC is known to induce neurotoxicity and is associated with structural and behavioral changes, including emotional and cognitive function. Furthermore, physiological, biochemical, and psychological effects responsible for euphoria, relaxation, and hypothermia induced by Δ^9^-THC are due to dybenzopyrane derivative. Neuropharmacological characterization revealed that Δ^9^-THC could induce catalepsy in mice. Later studies presented the chemical structure of Δ^9^-THC, a highly hydrophobic compound, and it was presumed that the Δ^9^-THC prominent effect is the modulation of cell membrane fluidity rather than acting on cell surface receptors [69,70]. Although Δ^9^-THC is not an endocannabinoid, it is a highly targeted topic in our current society due to the fact that it may have some medical benefits while being labeled a narcotic [67]. Like AEA, Δ^9^-THC binds to CB_1_ and CB_2_ receptors as a partial agonist and is associated with the receptor’s activation and in the regulation of downstream signaling molecules [67]. Δ^9^-THC is a lipophilic molecule, and it is not highly selective for receptors due to its partial agonistic characteristics [67]. Therefore, Δ^9^-THC will bind non-specifically at the site containing CB_1_ receptors [67]. The psychological changes with the use of Δ^9^-THC by a different route of administration, as normally seen with the use of recreational consumption of cannabis, are well established [71]. The psychoactive effect of cannabis is primarily caused by the inhibition of adenylate cyclase (AC), which in turn stops producing cAMP [67]. The reasons for the existence of medicinal marijuana is because Δ^9^-THC has some analgesic effects on patients and increases appetite [72]. If Δ^9^-THC is taken via inhalation, it enters the bloodstream through the alveoli and then enters the brain through the Blood–Brain Barrier [14]. The analgesic effect may occur when Δ^9^-THC binds to specific CB_1_ receptors on the neurons of the Periaqueductal Gray [14]. Δ^9^-THC, like other cannabinoids, will change the neurotransmitter release and suppresses neuronal excitability through hyperpolarization [14]. Δ^9^-THC exerts a pain-relieving effect from an acute and chronic condition (discussed in detail later). The presence of CB_1_ receptors in the hypothalamus has been proven to be responsible for the increased appetite upon Δ^9^-THC administration to rats. The CB_1_ receptor is responsible for increasing ghrelin, a hunger hormone normally released prior to a meal [72]. Once the digested food enters the duodenum, hormones called cholecystokinin and leptin are released [72]. The release of leptin reduces the effect of the CB_1_ receptor [72]. The hunger effect of Δ^9^-THC is reduced once the satiety signal is passed back to the hypothalamus [72]. Preliminary studies suggest that CB_1_ receptors associated with Δ^9^-THCs may induce dopamine during eating, which may explain the substantially increased desire to consume food [72]. Therapeutic advantages of synthetic analogs of Δ^9^-THC, including nabilone, in vomiting and nausea in 1981, dronabinol as an antiemetic in 1985 and appetite stimulant in 1992, and naviximols containing psychoactive Δ^9^-THC and non-psychoactive CBD, which was approved in 2005 as an effective drug for the treatment of pain in patients with MS and patients with advance cancer as well as spasticity associated with MS, are well described [73,74]. The role of other non-psychoactive cannabinoids in the modulation of Δ^9^-THC function is not conclusive and both inhibitory and activation functions are proposed. Furthermore, studies have also described the synergistic effect of Δ^9^-THC with opioids (discussed in a section of Heterodimerization). In addition to Δ^9^-THC, several other plant extracts, which are known to govern significant medicinal or clinical values are shown in Figure 4 and are discussed briefly here.

### 3.2. Cannabidiol (CBD)

In addition to the Δ^9^-THC, the second Phyto-CBs that draws significant attention to understanding the mechanism of action of cannabis is Cannabidiol (CBD). CBD is a major non-psychoactive cannabis with valuable therapeutic implications in many pathological conditions including antioxidant, anti-inflammatory, anti-convulsant, anti-depressant, anti-psychotic, anti-tumor and neuroprotective roles in several neurological diseases as well as an effect on post-traumatic stress disorder. The CBD effect is not mediated by CB1R, and studies have shown that CBD is devoid of CBRs binding and serves through the different targets, including as an agonist to TRPV 1 and 2 and as an antagonist to orphan GPCR, namely GPR55 [21,75]. Unlike Δ^9^-THC, CBD interacts with serotonergic and adenosine mediated signaling and poses complex pharmacology [76,77]. Earlier studies also support the role of CBD as a non-competitive antagonist for CB1R and inverse agonist for CB2R, in addition to the inhibitory role on AEA degradation and agonist activity for serotonin receptor HT_1A_. CBD exhibits activity on GPR18 and FAAH enzymes involved in cannabinoid metabolism in addition to working as an allosteric modulator and blocking CB1R-mediated signaling. CBD is an FDA approved drug for epilepsy involving GABA-A and adenosine A1 receptors [78,79].

### 3.3. Cannabidiolic Acid (CBDA)

CBDA is a non-psychoactive cannabinoid and precursor of CBD. It seems that the medicinal value, if there is any, for CBDA, has not been explored because CBD is a natural derivative of CBDA. CBDA is known for relieving nausea and inflammation and several folds more effective than CBD in the regulation of nausea and vomiting. Although, CBDA mediated prominent effects including anti-inflammatory and anticancer preferences are at primitive stage, CBDA and Δ^9^-THC are more effective in combination at doses that are ineffective in a single use.

### 3.4. Cannabigerol (CBG)

CBG exhibits low affinity for CB1R and CB2R and works as a weak agonist for TRPV1 and 2 and also blocks the 5HT1A receptor as well as works as an agonist to α-2adrenoceptors. Furthermore, in vitro studies have also demonstrated that Peroxisome proliferator-activated receptor-α (PPAR-α and -γ) are activated in the presence of CBG [80]. CBG has been used as an analgesic, anti-inflammatory, intraocular pressure suppressor, antibacterial, appetite stimulant, neuroprotective, bone growth promotor, and vasodilator and is also beneficial for relieving depression.

### 3.5. Cannabinol (CBN)

A non-enzymatic oxidation of Δ^9^-THC resulted in the production of CBN with higher concentration in older plants [15]). CBN has been extracted in good amounts from plants and exhibit its own activity and is known to have anti-insomnia, antibacterial, appetite stimulant, and pain relief properties. Previous studies have shown that, like CBD, CBN has the ability to inhibit the metabolism of aminopyrine and morphine through interfering with hepatic enzymes [81]. Also, CBN is known to be involved in the depression of intestinal motility [82] and has effects on corneal areflexia, as well as working as a weak anticonvulsant [83,84]. Like Δ^9^-THC, most effects of CBN are believed to be through ECS. Although not well established yet, CBN is well known as a sleep-enhancing cannabinoid.

### 3.6. Cannabichromene (CBC)

CBC is a major phytocannabinoid constituent of the cannabis plant. CBC is known to have a crucial role in brain function and neurogenesis. In addition, studies have also described antiviral effects, also known to work as an analgesic, antidepressant, antinociceptive, and anti-inflammatory. However, the mechanism involved in such diverse roles of CBN is not well defined except poor efficacy to CBR subtypes. CBC is a cannabinoid that serves as CB_2_ receptor agonist [85]. Udoh et al., further emphasized that CBC function as a selective agonist to CB2R and induced hyperpolarization in AtT20 cells more effectively than Δ^9^-THC and also proposed a role in CB2R dependent manner [85].

## 4. Synthetic Cannabinoids

Later, the identification of Phyto-CBs and observations with medicinal potential prompted the synthesis of several structurally different analogs, amongst them CP55940, WIN 55212-2, and JWH-18, which are known agonists to CBR subtypes. The structure of these synthetic analogs are shown in Figure 5.

Like endogenous and Phyto-CBs, synthetic cannabinoids also act through CB1R and CB2R. The majority of these compounds are potent agents for CB1R and exhibit higher affinity than Δ^9^-THC. Recent studies have shown that some synthetic cannabinoids in comparison to THC or cannabidiol, are lacking therapeutic abilities and rather exhibited severe cognitive and amplified psychiatric abnormalities. Also, synthetic cannabinoids are highly potent and are preferable recreational drugs due to their strong psychoactive effects, and these drugs are undetectable in serum and urine test. Furthermore, associated toxicity and death pose major threats to society and the healthcare system. Synthetic cannabinoids displayed significant variation in their effect that are possibly related to individual drug properties, route of administration, and doses, as well as, importantly, the lipophilicity of the compound. However, many synthetic cannabinoids have been discontinued from therapeutic use due to their binding to CB1R and unwanted adverse effect in CNS. Castaneto et al., described several fold more pharmacological effects of synthetic cannabinoids than Δ^9^-THC in several pathological conditions including anticancer growth effects, anti-seizure, weight-loss, anti-inflammatory, and analgesic effects [86]. Some of the synthetic cannabinoids including dronabinol and nabilone according to US Food and Drug Administration are approved for the treatment of nausea and vomiting in cancer patients. In the case of multiple sclerosis (MS), a drug of combination of equal amount of Δ^9^-THC and cannabidiol known as Sativex (nabiximols), although not supported highly, is used to treat spasticity. The use of synthetic cannabinoids through binding to CB2R and modulation proinflammatory processes and regulation of astrocytes in different conditions has recently been appreciated. Studies have also supported the role of synthetic cannabinoids independent of ECS; however, the molecular mechanisms involved are not well understood. Although, the uses of synthetic cannabinoids are promising in many pathological conditions, but the beneficial effects are not spared from adverse and unexpected side effects.

## 5. Cannabinoid Receptors: Structure, Function, and Distribution

Cannabinoids, like many other agonists for other GPCRs coupled to Gi, inhibit enzyme AC, which suggests that cannabinoid receptors (CBRs) belong to the GPCR family. To date, at least two CBRs, the type 1 (CB1R) and type 2 (CB2R) receptors, have been described with regard to their primary structure, ligand-binding properties, and signal transduction systems [15,87,88]. CB1R and CB2R are a member of the class A GPCR family. Based on anatomical distribution, CB1R is a prominent receptor with widespread distribution in different brain regions. In the mammalian brain, it is believed that CB1R is the highly expressed Gi/Gq coupled receptor [15,89]. CB2R is mainly expressed in peripheral tissues (discussed later in detail) [90]. Importantly, CBR subtypes expression in oligodendrocytes in the white matter of certain brain regions have drawn significant attention [91,92]. Several studies support the role of CB1R in the modulation of excitability and neurotransmitters release. In different target tissues, CBRs are involved in various functions, including inhibition of Ca^2+^ current, increased A type of K current, release of Ach from myenteric plexus, GABA release from hippocampal slices, and release of norepinephrine from the sympathetic nerve. CBR subtypes share >48% sequence similarity, couple to Gi/o inhibitory protein, inhibit AC and calcium channels, stimulate K channels, and are involved in the regulation of multiple signal transduction pathways are discussed in detail later.

### 5.1. Cannabinoid Receptor 1

Human chromosome 6 contains the gene CNR1, which is responsible for encoding CB1 receptors [12,93] described as a member of the GPCR family with 473 amino acids (AA) from rat brain cDNA and named CB1R. Further studies progressed and identified human (472 AA) and mouse (473 AA) homologs with 97–99% sequence similarity [22,94]. The splice variants have been reported for CB1R and proposed to exhibit distinct binding than full-length receptors. These two N-terminal splice variants displayed low expression in comparison to the main isoform [95,96]. Although controversies exist, polymorphism has been reported in CB1R that is associated with different pathological conditions, including obesity, schizophrenia, attention-deficit/hyperactivity disorder in children and depression linked to Parkinson’s disease [97,98,99,100]. In CNS, CB1R is the receptor subtype that exerts majority of biological effect of cannabinoids and is believed to be responsible for most psychoactive activities with the use of Δ^9^-THC [101,102]. The CB1 receptor is a typical GPCR with seven transmembrane domains. It is approximately 473 amino acids long with typical seven membranes heptahelical structure containing 3 intracellular and 3 extracellular loops and extracellular N-terminal intracellular C-terminal tail [14]. Moreover, molecular details of structure and function studies revealed that glycosylation of CB1R resulted in an increase in molecular size from 53 kDa to 64 kDa, including in preparation from the human brain [103,104]. Consistent with the existing concept of GPCRs posttranslational medication, palmitoylation at Cys^415^ at the C-terminal of CB1R is reported and that function in receptor anchoring to lipid raft at the cell membrane [105]. Studies using the mutagenesis approach identified the transmembrane helix as a binding site. Depending on the co-expression of CB1 receptors with other GPCRs, and exist as oligomers, heterodimers, or even homodimers (discussed in detail under section Dimerization).

The CB1 receptors expression is not restricted to CNS and peripheral tissues but has also been reported in vascular endothelium and the presynaptic terminal part of glutaminergic and GABAergic interneurons [106]. As a result, CB1 receptors can play a role as a neuromodulator, inhibiting the release of glutamine and GABA [106].

### 5.2. Cannabinoid Receptor 1 Distribution and Functions

The observations describing the suppression of cAMP in cultured neuroblastoma cells and proposing the involvement of Gi-coupled receptor was the first indication regarding the expression of CBRs [107,108,109]. Devane et al., using immunohistochemical and radioligand binding techniques, mapped brain CBR subtypes [110]. Hykeham et al., for the first time, described the distribution of CB1R in rat brains using quantitative autoradiography [111]. CB1R is well expressed in a different type of neuronal population in different brain regions with expression in axons, dendrites, and cell bodies. Quantitative autoradiography was executed by radiolabeling using a synthetic CB1R agonist called ^3^H-CP55,940 and by allowing the agonists to bind to CB1R [111]. Results indicating the presence of CB1R at presynaptic terminals with abundant expression of receptors in the cerebral cortex, hippocampus, basal ganglia, globus pallidus, and dopaminergic-rich midbrain regions including the substantia nigra, spinal cord, entopeduncular nucleus, and regions of the cerebellum and are responsible for multiple functions in CNS [111]. CB1R is primarily expressed in neuronal cells of presynaptic terminals. This distributional pattern of CB1R explains the hindered mobility and psychoactive effect of Δ^9^-THC [111,112,113]. In addition, CB1R expression has been reported in limited peripheral tissues with unique functions. Comparative distribution of CB1R density in different brain regions is summarized elsewhere [59]. Studies also indicate that CB_1_ receptors are in low concentrations in regions of the brainstem, such as the medulla and pons [111]. Because medulla oblongata and pons are mostly responsible for the respiratory and cardiovascular systems, rats exposed to Δ^9^-THC had normal respiration and heart rate [14]. Quantitative electron microscopic observations provided evidence that the majority of the CB_1_ receptors were located in the terminal ends of the axons and along the axons themselves [114]. Also, it showed small amounts of CB_1_ receptors on the dendrites, proximal axons, and cell bodies [114]. Like many other GPCRs, CB1R was confined to the cell surface;; however, CB1R association with the nucleus has also been reported earlier [115]. These results indicate the potential effect of cannabinoids on neurotransmission.

CB_1_ receptors coupled to G_i_ and G_o_ proteins and are presynaptic receptors that regulate many other neurotransmitters. CB_1_ receptors can be activated by endocannabinoids or introduced into the body by either cannabis or a similar synthetic cannabinoid [116]. Ultimately, when CB_1_ receptors bind to cannabinoid ligands, AC is inhibited from producing cAMP, voltage-gated Ca^2+^ channels are inhibited, mitogen-activated protein kinase (MAP kinase) are activated, and inwardly rectifying potassium channels are activated [116]. These voltage-gated Ca^2+^ channels and inwardly rectifying potassium channels are activated by proteins called Protein Kinase A and Protein Kinase C [116]. Furthermore, these protein kinases are activated by cAMP, which in turn are produced by AC [116]. Although, the binding of CB1R to Gi is well established, depending on some conditions, it has also been reported that receptor couple to Gs may influence the formation of cAMP and signal transduction pathway differently.

Immunocytochemical studies showed high levels of CB_1_ receptors in GABAergic interneurons and glutamatergic terminals [117]. GABA, commonly known as γ-Aminobutyric acid, is an inhibitory neurotransmitter that induces hyperpolarization to inhibit signal transmission [106]. Conversely, glutamate is the most prominent excitatory neurotransmitter in the human body [118]. Of course, depending on the location of the brain, there is a different abundance of GABAergic and glutamatergic neurons. The finding of these pathways proves that CB_1_ receptor stimulation inhibits neurotransmitter release and suppression of excitability by depolarization [116]. Although controversial, the expression of CB1R has also been reported in mitochondria [119,120]. CB1R has the ability to sequester G protein, and consequently, CB1R is not available to couple another receptor [90]. The study showed the loss of SST and NE mediated inhibition of Ca^2+^ by pertussis toxin-sensitive Gi/o couple receptor of NE and SST by expression of hCB1R in superior cervical ganglion neurons but not by mGluR2 [90]. Recent studies have also shown colocalization of CB1R with SST and brain nitric oxide synthase (bNOS) in the rat brain hippocampus and hypothalamus, respectively [121,122]. Furthermore, studies have shown the colocalization of CB1R with several other members of the GPCR family in different brain regions with significant functional consequences (discussed later in detail). As proposed earlier, understanding the precise localization of CB1R might play a crucial role in selective targeting and minimal adverse effects when used as a therapeutic alternative [35,123,124].

### 5.3. Cannabinoid Receptor 2: Distribution and Functions

The CB_2_ receptor, a typical GPCR with seven transmembrane domains, is approximately 360 amino acids long and coded by the CNR2 gene located on chromosome 1p36 [13]. CB2R shared 44% structural similarity with CB1R;; however, when only considering the transmembrane regions, the two sequences are 68% similar, whereas S3.31 and F5.46 are two residues of CB_2_ receptors that distinguish it from CB_1_ receptor [13]. Furthermore, studies also support that upon ligand binding, CB2R S3.31 and F5.46 residues form hydrogen bonds resulting in conformational changes, which trigger signaling pathways [13].

Like CB1R, the activation of CB2R receptors resulted in the inhibition of AC and cAMP formation [87]. However, CB2R can also be coupled to the MAPK–ERK pathway [125]. The function of CB2R depends on the type of ligands bound to the receptor. For instance, CB_2_ receptors function to inhibit AC, and this occurs once the CB2R is activated in the presence of noladin or CP-55,940, whereas the MAPK–ERK pathway is activated in the presence of 2-AG [125].

**Figure 6:**
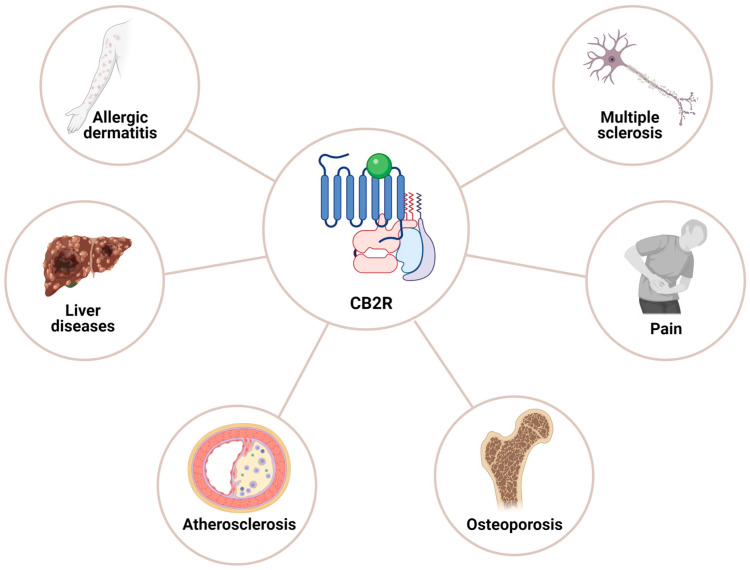
Role of cannabinoid receptor 2 (CB2R) in different pathological conditions. This figure was created with BioRender.com.

CB2R displayed a distinct role in target tissues once activated either by 2-AG or receptor-specific agonist (JWH015 or JWH133), including the inhibition of locomotion [125,126], morphine-6-glucuronide-induced emesis [126], and neuropathic pain [127,128]. The neural progenitor cell proliferation, neuroprotection, and inhibition of neuronal firing in the dorsal root ganglia and spinal cord and GABAergic transmission in rat cerebral cortex have also been reported [129,130,131,132]. Xiong Xi et al., further emphasized that CB2R in a dopamine-dependent manner is involved in the modulation of cocaine-associated reward and locomotor effects [132]. CB2R-mediated regulation of signaling pathways including AC, cAMP, and PKA, as well as ERK1/2, P38 and JNK is associated with certain cell physiological functions such as cell proliferation, differentiation, and survival. In addition to CB2R coupling to Gi/o, CB2R also couples to Ca^2+^ via inositol triphosphate receptors and regulate neuronal excitability through activated Cl channels. Moreover, the prominent role identified for CB2R is the modulation of neuronal and glial cells function and consequent implication in neuroprotection by inhibiting the factors associated with neurotoxicity (Figure 6). It should also be noted that the most beneficial effects of CB2R agonists if used as a therapeutic alternative in any given pathological conditions exert no psychotic effects.

CB2R is a prominent receptor of the peripheral system with high expression in immune cells. However, the concept that CB2R expression is only limited to the peripheral tissues has recently been challenged and studies have demonstrated CB2R expression relatively at low levels in different regions of the normal brain [13]. Moreover, the functional role of CB2R in different brain regions is still not well described contrary to its role in peripheral tissue, including in microglia. Growing evidence supports that CB2R is expressed in different brain regions relatively less than CB1R. Previous studies show that CB2R-like immunoreactivity in brain regions including the cortex, hippocampus, cerebellum, olfactory lobe, substantia nigra, thalamus, hypothalamus, spinal cord, and medulla is expressed in a region-specific manner [23,126,133,134]. CB2R expression at the level of protein in microglia at the perivascular vicinity in cerebellar white matter as well as in astrocytes of human fetal brain tissues has been reported [135,136]. Ashton et al., also demonstrated CB2R in fibers of the granular layer, which was lacking colocalization with GFAP that indicates the possibility that these fibers are microglial or possibly neuronal [137]. Subcellular distribution revealed CB2R-like immunoreactivity in the soma, neuronal process, and glial cells. Based on region-specific distribution in CNS, different roles for CB2R in the brain have been assigned. Taken into consideration, these results summarized the presence of CB2R in the brain at the level of protein and mRNA using immunohistochemistry, Western blot analysis, and RT-PCR. In the normal healthy brain, microglia are devoid of CB2R expression, but conditions such as neuroinflammation have the ability to induce CB2R in these cells [138,139]. Femke S den Boon et al., demonstrated the presence of CB2R in pyramidal neurons of prefrontal cortex layer II/III and defined the role in Ca^2+^-activated Cl channels in am IP3 R-dependent manner suggesting CB2R-mediated regulation of neuronal excitability [46]. However, one of the significant observations includes the increased CB2R microglial expression in neurological condition as well as CB2R-mediated regulation of microglial activation in response to endocannabinoids. These observations attest to the crucial contribution of CB2R in neurological diseases associated with neuroinflammation and primary inflammatory diseases such as MS.

CB_2_ receptors have also been detected in immune cells such as B cells, T cells, macrophages, neutrophils, and mast cells [129]. It should also be noted that CB2R presence has also been reported in neuritic plaque-associated glia in Alzheimer’s disease brain [135,140,141], human brain capillaries, and microvessels [142], as well as in a model of encephalitis induced by simian immunodeficiency virus [143]. The presence of CB2R in human astrocytes not only indicates the role of the receptor in pathological condition but further emphasizes the expression of CB2R in the normal brain [23]. Stempel et al., for the first time, demonstrated the presence of CB2R in CA2 and CA3 hippocampal regions and described the role of CB2R in neuroplasticity [144]. These observations further emphasized the functional association between cannabinoid-mediated signaling and transporter for sodium bicarbonate. The observations by Stempel et al., further suggested non-overlapping functionality between CB1R and CB2R at the cellular level and proposed the presynaptic expression of CB1R with postsynaptic CB2R in pyramidal neurons of the CA3/2 region of the hippocampus [144].

## 6. Cannabinoid Receptors Internalization

Most, if not all, members of the GPCR family are often upregulated at the cell surface or internalized in response to agonist treatment in a time- and temperature-dependent manner. Moreover, during the process of internalization, some receptor proteins recycle back to the membrane, whereas others are sorted out and end up in lysosomes for degradation that results in loss of receptor functionality at the cell surface due to less available receptors [145,146]. However, sustained and prolonged exposure to agonists leads the receptor desensitization in contrast to agonists, which induces a wide variety of changes. Interestingly, in the case of GPCRs, which often induce psychotic effects, tolerance, dependence, and addiction, they respond in a distinct manner. Like many other GPCRs, prolonged activation of CBRs in the presence of agonists plays a crucial role in receptor-mediated signaling pathways. To understand the significance of CBRs in pathological conditions, it becomes essential to understand receptor internalization, desensitization, and recycling back to the cell surface or targeting for degradation. In this context, previous studies have shown great diversity in the efficacy of different agonists of CBR trafficking. The endogenous and exogenous agonists of CBRs work in a time-dependent manner. CB1R and CB2R both exhibit a different rate of internalization depending upon the source of expression.

### 6.1. Cannabinoid Receptors 1 Internalization

Long-term exposure of CB1R to the agonist resulted in receptor desensitization and eventual loss of receptor at the cell surface. Several earlier studies have shown the internalization of CB1R and its recycling back to the cell surface in the absence of agonist. Furthermore, this ability of CB1R is not only restricted to neurons expressing the receptor endogenously but was also reported in heterologous expression systems [147,148,149,150]. A CB1R with a mutation in the second transmembrane domain lacks recycling back to the cell surface; however, such observation could not be reproduced by Canals and Milligan using the same mutation. In addition, such controversial observations have also been reported in some other studies in the presence of ligands [147,151,152,153]. The role of different proteins, including GRKase, β-arrestin, and sorting proteins involvd in receptor internalization and desensitization, is crucial. Recent studies suggest the role of clathrin-coated pits in the internalization of CB1R upon binding to agonists [150,154,155,156,157,158]. Although, the role of β-arrestin-2 in the internalization of many GPCRs and other receptor endocytosis is well established, its contribution in CB1R internalization is not well understood. Moreover, the internalization and recycling of constitutively expressed CB1R have been proposed in a β-arrestin-dependent manner [147].

### 6.2. Cannabinoid Receptors 2 Internalization

CB2R is the prominent receptor and holds some promises regarding therapeutic implication. Unlike CB1R, the use of CB2R activation is devoid of psychotic effects. CB2R is neuroprotective and exerts a neuromodulatory role [159] and is active in the treatment of acute as well as neuropathic pain. Repeated and prolonged exposure of GPCRs, including CBRs, accounts for the changes in receptor response compared to short-term activation. Although not well studied yet, CB2R has shown diversity in internalization but often exhibits basal internalization due to constitute activation. Atwood et al., characterized the role of agonists and inverse agonists on CB2R internalization [159]. It seems that the internalization of the receptor is a critical determinant of the functional activity of the agonist. Agonist-dependent internalization of CB2R that is blocked in the presence of an antagonist has been reported [160]. Atwood et al., demonstrated a distinct response and diversity of cannabinoid ligands on CB2R internalization and proposed that a synthetic cannabinoid, often referred to as nonclassic cannabinoid, is more effective in the induction of internalization [159]. Despite the inhibition of forskolin-stimulated second messenger cAMP and internalization of CB1R, WIN55,212-2 is unable to prompt CB2R internalization, whereas the blockade of CB2R internalization occurs in response to CP55 [156,161,162].

## 7. Endocannabinoid Retrograde Signaling and Synaptic Plasticity

In the CNS, eCBs are known as a prominent modulator of synaptic function. The concept that endogenous cannabinoids are released from postsynaptic neurons on demand and act on CBR subtypes, which are located on presynaptic sites and function in a retrograde manner, is well established [32,163] (Figure 2A). Following release from the plasma membrane endocannabinoids, AEA and 2-AG act in a autocrine and paracrine manner and modulate CBR subtype-mediated signaling [164]. The presence of CB1R in axon terminals in the hippocampus involves in regulation of the release of GABA in response to eCBs and is followed by studies showing the release of multiple neurotransmitters, including glutamate, serotonin, and dopamine in different brain regions [165,166]. The functional consequences of such profound retrograde signaling were first described with the changes at inhibitory and excitatory synapses in an activity-dependent manner, including short and long-term plasticity. This was first reported in the hippocampus and cerebellum in response to the depolarization of postsynaptic neurons. Depolarization exerts transient suppression of inhibitory synaptic input known as depolarization-induced suppression of inhibition (DSI), also depolarization-induced suppression of excitation (DSE) [32,167,168,169]. The process of DSI involves the role of released eCB from the postsynaptic terminal, acting on CBR expressed on presynaptic terminal inhibitory interneurons and resulting in suppression of firing and the release of the inhibitory neurotransmitter GABA. However, DSE is depolarization-induced suppression of excitation that is associated with the release of the excitatory neurotransmitter glutamate. Katona et al., showed the expression of CB1R at the neuronal terminal and proposed a crucial role in the regulation of synaptic function [170]. Cannabinoid antagonists AM281 and SR141716A abolished cannabinoid agonist WIN55212-2-mediated suppression of GABA at DSI positive synapses from presynaptic terminals but not from the synapsis devoid of DSI [167,171]. Furthermore, studies also support that Ca^2+^ release induced via flash autolysis is able to induce DSI [168]. The role of the released cannabinoid from the postsynaptic terminal is limited at inhibitory terminals and observed in excitatory presynaptic fibers of cerebellar Purkinje cells following depolarization and referred to as depolarization-induced suppression of excitation (DSE). DSE, which lasts for a very short time, is blocked by the Ca^2+^ chelator BAPTA and cannabinoid antagonist, indicating inhibition of glutamate release due to activation of CB1R [169,171]. In addition to the crucial role of CBR, these studies also highlight the prominent contribution of voltage-gated Ca^2+^ channels in DSI and DSE. Furthermore, it is not surprising to note that DSI and DSE are taking place in other brain regions including the hippocampus and cerebellum, displaying strong CBR expression with a significant physiological function [27,172]. Furthermore, the subcellular distribution of metabotropic glutamate receptor 5 (mGluR5) and muscarinic receptor 1(MR1) in medium spiny neurons in the striatum is associated with the modulation of 2-AG-mediated retrograde signaling. Uchigashima et al., revealed that activation of mGluR1 enhanced DSI and DSE, whereas MR1 activation resulted in a stimulatory effect on DSI only, which was blocked in the presence of DAG lipase inhibitor and proposed that 2AG is a major eCB in striatal medium spiny neurons to induce retrograde signaling [173].

eCBs mediated synaptic function, and signaling is not only limited to the activation of CBR subtypes; the role of TRPV1 channels and indirectly via interaction of neuronal and glial cells has also been proposed (see for details reviews [32,55,138,171,174]. TRPV1 channels, which are prominently involved in regulating pain sensation-associated synaptic transmission, are expressed in afferent peripheral sensory neurons. AEA serves as a partial agonist to CB1R and has the ability to function as a full agonist to TRPV1, which is also expressed in CNS and participates in synaptic transmission [32]. Furthermore, studies have shown that the synthesis of AEA is prompted by activating mGluR5 via phospholipase C (PLC) and releasing Ca^2+^ and, consequently, activating TRPV1 [175]. Activated TRPV1 mediates LTD specifically in D2R-expressing neurons in different brain regions, including the nucleus accumbens, dentate granule cells, and bed nucleus [58,176,177]. In addition to TRPV1, the role of GABAergic interneurons in nonretrograde signals is also reported, which involves post-synaptic hyperpolarization in a CB1R-dependent manner, which further suppresses its suppression excitability [178]. eCB-mediated signaling has also been described beyond neuronal cells, and studies have shown that astrocytes, oligodendrocytes, and microglial cells have the ability to synthesize eCBs [179,180]. While the role of eCBs in astrocytes and synaptic plasticity is emerging, it is not certain for glial cells. From the discussion above and in view of growing evidence, it seems that retrograde signaling is the possible method of postsynaptic response in regulating presynaptic activity, as proposed earlier [171].

## 8. Cannabinoid Receptors and Signal Transduction Pathways

The pharmacological and physiological impacts of CBR activation depend on and are mediated by several downstream signaling pathways in different target tissues. It is believed that activation of CBRs in CNS might be distinct from peripheral or endocrine systems. CB1R and CB2R belong to the transmembrane GPCR family and are coupled to the PTX-sensitive G_i/o_ protein and suppress AC and the formation of cAMP following agonist-dependent activation of the receptor [15]. However, this G protein coupling of CB2R is not restricted to the G_i/o_ protein only, and other G proteins are also activated by CB2R, which depends on the ligand and type of the cells used [181]. Although CBR subtypes prominently bind to Gi, few studies have also proposed receptor coupling to Gs [182,183,184]. In contrast to Gi coupling, relatively, at lower doses of cannabinoids CBR subtypes couple to Gs and mediate an effect that is linked to increased AC and cAMP as well as increased intracellular Ca^2+^. Furthermore, such stimulatory effects also resulted in aggressive behavior, hyperalgesia, increased motor activity, and increased body temperature. Recent studies have also described switching of receptor coupling from Gi to Gs during the process of CBR subtypes heterodimerization with other members of the GPCR family. To ascertain the molecular mechanism and functional consequences of CBR coupling to Gs, it needs to be revisited to understand whether this change holds some pharmacological significances.

## 9. Central Nervous System and Cannabinoid Receptors Mediated Signaling Pathways

The pharmacological characterization of CB1R following ligand binding and the regulation of multiple downstream signaling pathways is now well established despite a limited understanding of the molecular details of how the ligand binds to the receptors at the cell surface. CB1R is able to stimulate specific AC isoforms via Gβγ subunits of G proteins [26,185]. Also, the CB1R in a complex with dopamine receptor 2 (D2R) stimulates cAMP via coupling to G_s_ in cultured striatal neurons, and in experiments when Gi is blocked by PTX in transfected CHO-K1 cells, as well as in rat globus pallidus slices preparation upon treatment with relatively high concentration of WIN 55, 212-2 (WIN) [26,183,186,187]. However, no increase in intracellular Ca^2+^ concentration via G_q/11_ protein has been shown in transfected HEK-293 cells and cultured hippocampal neurons with endogenous receptor expression in presence of any other CB1R agonists except the same concentration of WIN [26,188]. Moreover, CB1R endogenously expressed in astrocytes and in slices prepared from mice hippocampus is coupled to G_q/11_, increases intracellular Ca^2+^ concentrations, and triggers the astrocytic release of glutamate that activates the N-methyl-D-aspartate receptor (NMDAR) on pyramidal neurons, and it is indirectly involved in synaptic transmission [26,189].

Previous studies have shown CB1R-mediated modulation of several types of ion channels activity, including the inhibition of the N-type Ca^2+^ channel in neuroblastoma as well as by using cultured hippocampal neurons and cerebellar slices prepared from mice brains [26,187,190]. At present, the concept that CB1R regulates Ca^2+^ influx to inhibit GABA release in mouse hippocampal slices via modulation of the activity of presynaptic N-type Ca^2+^ channel is well established [26,191]. Furthermore, negative regulation of other types of Ca^2+^ channels, including P/Q-type, and R-type Ca^2+^ channels, have also been shown in presence of activated CB1R in various systems [26,192,193,194,195]. CB1R regulates the activity of G protein-coupled inwardly rectifying K^+^ channels (GIRKs) as well [195,196,197]. Also, CB1R activates GIRK in slices prepared from mouse nucleus accumbens, and in rat sympathetic neurons injected with CB1R complementary deoxyribonucleic acid (cDNA) as well as in AtT-20 cells transfected with CB1R [26,195,196,197].

The activation of CB1R either in cells with constitutive receptor expression or in transfected cells involved in the regulation of cell proliferation, cell cycle control, and cell death through the activation of mitogen-activated protein kinase (MAPK) signaling pathways, including extracellular signal-regulated kinase 1/2 (ERK1/2), c-Jun N-terminal kinase (JNK), and p38 [26]. Growing evidence supports CB1R-mediated regulation of MAPK signaling in a cell-type and ligand-specific fashion [181,198,199]. This also should be noted that CB1R-induced ERK1/2 activation can be mediated by a G protein, β-arrestin, or PI3K, heavily dependent on the microenvironment and stimulus type [26,200,201,202]. Comparable observations have also been made regarding the activation of p38 following stimulation of CB1R in different experimental conditions including human vascular endothelial cells, transfected CHO-K1 cells, and rat/mouse hippocampal slices [26,203,204,205]. In addition, JNK activation has been shown in transfected CHO-K1 cells, where G proteins, PI3K, and Ras were involved in the transduction [26,204]. Moreover, the activation of JNK in Neuro2A cells exhibiting endogenous expression of CB1R was also observed and was proposed that it may be related to CB1R-mediated neurite outgrowth [26,206]. In brain regions, including the striatum, nucleus accumbens, and hippocampus; CB1R-mediated increased ERK1/2 activation is shown following acute treatment with Δ^9^-THC in comparison to increased ERK1/2 protein expression in the cortex and hippocampus upon chronic treatment [207,208,209]. Daigle et al. [210] proposed that increased ERK1/2 described by Derkinderen and Valjent [207,208] upon chronic Δ^9^-THC use is probably due to CB1R desensitization.

## 10. Cannabinoid Receptor-Mediated Regulation of Signaling in a G Protein-Independent Manner

In addition to G protein-dependent mediated signaling as normally seen with most GPCRs, the CB1R also has an ability to signal independent of G proteins through association with other molecules such as β-arrestin [199]. β-arrestin is a key mediator of GPCR desensitization. β-arrestin binds to the receptor post GRK-induced receptor phosphorylation and initiates the receptor internalization process, during which β-arrestin could mediate signaling pathways [211]. Furthermore, β-arrestin 2-dependent desensitization of the CB1R has also been demonstrated in various other systems [212,213]. Previous studies have shown that in β-arrestin 2-mediated desensitization, but not internalization, of CB1R in transfected HEK-293 cells is a crucial determinant for the duration of ERK1/2 phosphorylation following activation of CB1R [26,154]. Furthermore, follow-up studies established a positive correlation between the duration of CB1R interaction with β-arrestin at the cell surface in a ligand-specific manner and the extent of β-arrestin-mediated signaling [201]. The critical role of β-arrestin 2-mediated regulation of CB1R activity is further strengthened by using β-arrestin 2 knockout mice [214,215]. Despite an increased sensitivity to Δ^9^-THC, knockout mice devoid of β-arrestin 2 displayed a comparable expression level of CB1R featuring enhanced antinociception and decreased tolerance [26,214,215]. Furthermore, the role of β-arrestin 1 in the phosphorylation of ERK1/2, MAPK 1/2, and the proto-oncogenic tyrosine-protein kinase, Src, in response to a CB1R allosteric modulator, ORG27569, has been suggested in emphasizing a signaling mechanism that is mainly dependent on stimulus [26,216].

## 11. Role of Cannabinoids in Regulation of Cell Survival Signaling Pathways

In addition to the MAPK signaling molecules, the prominent regulator of cell proliferation, growth, and death is the PI3K/Akt signal transduction pathway. The CB1R has been implicated in activation of the PI3K/Akt pathway in human astrocyte cells, rat primary cultured astrocytes, and transfected CHO-K1 cells, with CB1R-induced protective effects on cell survival [200,206,217]. The CB1R in nutrient-deprived rat oligodendrocyte progenitors promotes cell survival and modulates cell differentiation via the PI3K/Akt pathway [26,91,218]. In parallel to these observation, HU-210, a CB1R selective agonist exhibited neuroprotection in rat cultured cortical neurons against the neurotoxin (S)-α-amino-3-hydroxy-5-methyl-4-isoxazole propionic acid through activation of the PI3K/Akt pathway but not MAPK pathways [219]. A previous study has demonstrated activation of the PI3K/Akt pathway, but not ERK1/2 in several brain regions following acute administration of Δ^9^-THC in mice [220]. Furthermore, CB1R-mediated protection was observed against excitotoxicity in *huntingtin* knock-in striatal neuronal cells via a PI3K/Akt signaling-mediated increase in brain-derived neurotrophic factor (BDNF) [221]. In addition, studies in the past have also described CB1R-mediated PI3K/Akt activation and modulation of oocyte maturation and embryonic development [222]. In addition, CB1R coupling to Gs and Ga/11 has also been reported in the modulation of distinct signaling pathways. However, the mechanism of CB1 and CB2 receptor coupling to G proteins is still a question of discussion. In this context, studies using single-particle cryoelectron microscopy and agonist-bound CB1R or CB2R with heteromeric Gi protein in order to determine the receptor structure are needed [223].

## 12. Morphological Changes in Central Nervous System with the Use of Cannabis

The observations regarding structural changes with the use of cannabis in the human brain are rather conflicting and data available are limited; however, animal studies revealed developmental changes with sustained and prolonged use of cannabis with psychotic effects in different brain regions with relatively high expression of CBRs. With the long-term use of cannabis, changes in the grey matter of cortical and hippocampal regions are reported, which are associated with perturbed motivational, emotional, and affective processing [224]. Studies have also reported changes in the cerebellum [225,226]. The morphological changes in CNS are associated with the amount of drug used and the level of dependence. Growing evidence with the use of cannabis at the age of adolescence signifies the structural changes in the brain with age. Also, a correlation between white matter damage and age has been found, and it is believed that the toxicity of drugs depends on binding to the target site. In contrast to these observations, multiple studies have also reported no changes in CNS with the use of cannabinoids [227,228]. Zalesky et al., reported impaired axonal connectivity in white matter-rich brain regions, including the hippocampus [229]. Most importantly, the exposure of the brain to cannabis for an extended time during development reduced the level of eCB and may induce apoptosis in oligodendrocyte progenitors and white matter development [91,225,230,231]. These observations emphasize the importance in teens and adolescents of the stage at which the use of cannabis begins. The morphological changes in the brain in respect to the relative expression of CB1R are not well established; however, they are directly correlated with the age of regular cannabis use [229]. Although, the results on human adolescent brain development with the use of cannabis are sparse, growing evidence from animal studies supports the vulnerability of the adolescent brain to the changes in ECS and its significant impact on developmental changes in CNS and occurrence of abnormal behavior. Taken together, the involvement of altered endocannabinoid signaling in adolescence is a critical determinant of not only behavioral changes but also in neurodevelopment. Whether the pattern of distribution of certain neurotransmitters or activity is affected in brain regions displaying a high expression of ECS in response to cannabis use by the adolescent and adult has not been discussed yet in detail.

## 13. Addiction, Withdrawal, and Cannabis Use Disorder: A Challenge or Limitation for Medical Use

Cannabis dependence has been proven by using animal studies and accumulated epidemiological results in humans. Growing evidence supports the development of tolerance with the chronic use of cannabis. Despite its significant beneficial role in many medical conditions, the use of cannabis has been reported to develop cannabis use disorder along with sever incidents of cannabis use disorder with addiction and withdrawal, especially with the use of Δ^9^-THC in addition to the potential psychological and behavioral changes [232,233,234]. It was proposed that a relative number of receptors and their function and cannabis-mediated plasticity in the brain is associated with tolerance. Furthermore, Martin et al., described that underlying cellular adaptation of tolerance is associated with the sequence of events involving G protein uncoupling from the receptor or desensitization and signal termination [154].

Repeated doses of Δ^9^-THC lead to psychological and analgesic effects and lead to tolerance through CB1R internalization [39,116,235]. In general, cannabis is devoid of a withdrawal system; however, the use of a CB1R antagonist in animals and humans is associated with withdrawal [116,235]. Murray et al., proposed that the withdrawal system does not probably appear due to the slow clearance of cannabis [235]. Recent studies have significantly contributed to our understanding of addiction following the use of cannabinoids. Like many other substances of abuse, cannabinoids are also involved in the regulation of dopaminergic transmission in ventral tegmental area, indirectly due to the presence on GABAergic and glutamatergic neurons and its release in the nucleus accumbens attesting to the role of cannabinoids in reward. Furthermore, cannabinoids-mediated modulation of GABAergic and dopaminergic systems in substantia nigra and mesocorticolimbic pathways is a sign of reward pathway and addiction. Changes in neuroplasticity following cannabinoid use possibly lead to dependence, addiction, and withdrawal. Interestingly, drug-seeking behavior and increased dopamine changes in the nucleus accumbens are prevented with the blocking of cannabis-induced signaling and CB1R, respectively. From the available data, it is concluded that cannabis plays a crucial role in addiction and withdrawal, possibly due to long-term neuroplasticity adaptation in response to a well-integrated manner via regulation of dopaminergic neurotransmission in the dopamine-rich brain region, including substantia nigra and mesocorticolimbic system. Contrary to the notion that use of cannabis is harmless and improves quality of life, multiple clinical and epidemiological studies argue in favor of addiction and induction or promotion of psychosis with the prolonged use of cannabis. From the preceding discussion, it is concluded that previous studies on the benefits and risks of cannabis have limitations. More extensive studies are needed to fully understand the medicinal use of cannabis and its potential benefits and risks.

## 14. Cannabinoids Therapeutic Use and Diseases

In recent years with the legalization of cannabis, several pharmaceutical organizations have been involved in synthesizing new cannabis derivatives showing clinical values and have an ability to afford beneficial effects in some pathological conditions. In parallel to the use of cannabis for recreational purposes, the use of cannabis is also on the rise for medical use. O’Shaughnessy proposed the use of cannabis for pain, vomiting, convulsion, and spasticity [236]. Currently, several clinical trials are underway to take advantage of the benefits of cannabis in certain medical conditions [237,238,239]. Many earlier studies support the role of CBR subtypes upon activation by eCB or exogenous addition of phyto-and synthetic cannabinoids in various pathological conditions including obesity/metabolic syndrome [240]; diabetes and associated complications [241]; neurodegenerative diseases [242,243], anti-inflammatory [244], cardiac complication [245,246,247], liver diseases [248,249], gastrointestinal [250], and skin diseases; [251] pain management [127,252]; neuropsychiatric disorders [253,254]; cachexia [255]; cancer of different origin [256,257]; and chemotherapy-induced nausea and vomiting [258], as well as many other complications. [35,255]. Furthermore, understanding the crystal structure of CB1R provides interaction with an antagonist as well as molecular details for binding of the ligand, including natural and synthetic analogs associated with psychoactive or protective effects.

The pathophysiological significance and therapeutic advantages of CB2R are much broader than CB1R subtypes [5]. In CNS, CB1R is the receptor subtype responsible for Δ^9^-THC-induced psychotropic effects and other side effects. The CB1R inverse agonists, rimonabant and taranabant, which have anti-obesity effects, are also associated with anxiety, depression, and suicidal tendencies, leading to their withdrawal from clinical use [5]. There is growing evidence in support for the role of CB2R agonists that have been implicated with considerable effectiveness in preclinical models of analgesia, inflammation, neuroprotection, anxiety, and ischemia/reperfusion injury; however, pathological significance and therapeutic exploration is still at a primitive stage [159]. Talarico et al., described cannabinoids as a new perspective for treating AD [259]. Although medical preparation of marijuana is lacking FDA approval, except limited cannabis analogs, the off-label use of cannabis in several pathological conditions is widely used. Accordingly, in the present review, whenever cannabis use is stated, it is in any given condition without any consideration of FDA approval or being off-label.

## 15. Cannabinoid and Its Relevance to Clinical Implication

**Figure 7:**
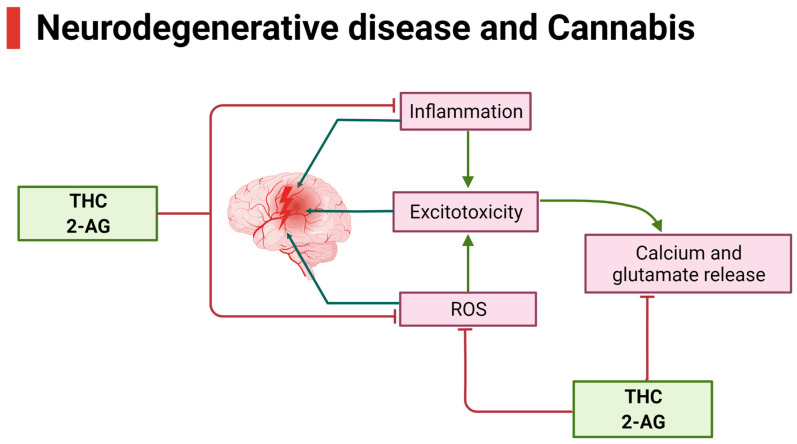
THC and 2-AG significantly suppressed the events in the brain associated with neurodegenerative diseases. This figure was created with BioRender.com.

Despite being designated as drugs of abuse, the therapeutic significance of cannabinoids is well accepted in several pathological conditions. Recent observations have placed cannabis as a neuroprotective compound, specifically Δ^9^-THC, with psychoactive properties and nonpsychotic cannabidiol. Increased expression levels of AEA and 2-AG in CNS during brain injuries are known to prompt eCB-mediated signaling pathways that afford neuroprotection through blocking complex events, including excitotoxicity, apoptosis, oxidative stress, and inflammation (Figure 7).

In several pathological conditions, as discussed above, including pain, obesity, inflammation as well as in addiction, CBRs serve as a potential therapeutic intervention upon activation in the presence of eCBs, Phyto-CBs, and synthetic analogs [88]. Three synthetic cannabis-based drugs, namely Dronabinol (Marinol^®^), a synthetic formulation of Δ^9^-THC and Sativex, a mixture of Δ^9^-THC and CBD in an equal ratio that alleviates spasticity in adult MS cases [14,15]. Nabilone (Cesamet^®^) synthetic derivative of Δ^9^-THC, is approved in 1982 for clinical use for the treatment of emesis [13].

Dronabinol was first licensed to control nausea and vomiting in patients going through chemotherapy as well as for appetite in acquired immunodeficiency syndrome (AIDS) patients. Walther et al., described the role of Dronabinol in AD and reported improvement in nocturnal motor activity and attenuation of agitation and aggression [260]. These results further support previous observations in elderly patients, including reducing anorexia and improving body weight and behavior [261]. Δ^9^-THC-mediated selective preservation of TH-positive neurons is also reported in an experimental model of PD [262]. Sativex has the ability to afford anti-inflammatory and antioxidant effects and has been used in the treatment of naturopathic pain and spasticity as well as sleep instabilities in MS patients. Studies have also reported the role of CB1R and CB2R in regulating the immune system in MS [237,263,264]. Sativex spray is approved for neuropathic pain in MS as well as cancer patients. Cannabis-based drugs have also been implemented in the treatment of Tourette’s syndrome and glaucoma, and a possible neuroprotective role in head injury, anti-inflammation, and some cases of cancer has been proposed. Taken together, these results, along with other information, support the potential role of cannabinoids in displaying disease progression and associated symptoms [265,266,267,268].

## 16. Early Use of Cannabinoids and Vulnerability to Neurological Diseases

The structural organization and neuronal functional connectivity of the brain begin at an early stage of development. These progressive changes do not end at birth but rather persist during childhood, in adolescence, and even at the stage of adulthood. However, regression in the morphological and transformational changes is highly expected in the presence of intrinsic and/or extrinsic insult and resulted in perturbed neuronal connectivity and impaired functionality. Moreover, such changes at an early stage resulted in dysfunctional memory and cognitive function. The use of cannabis commences at an early age as the brain develops. Previous studies have shown that ECS is implicated in multiple events involved in brain development, maturity, and function, including neural cell proliferation, migration, and differentiation [269]. Furthermore, enduring effects on cognitive functions, such as memory, attention, and reasoning have also been speculated with the prenatal use of cannabis. Despite the significant relevance of cannabis use during pregnancy on fetus development, including guiding the migration of neurons, synaptogenesis, and the formation of the blood–brain barrier, unexpected side effects have also been reported that were linked to impaired neurodevelopment. This is true for THC, which contains psychoactive ingredients and an ability to cross blood–brain barrier to exert direct effect on development fetus via activation of the CB1R subtype. Such changes involve synaptic plasticity, a process that is critical for learning and memory.

The exposure of the fetus to cannabis during embryonic development indirectly and the use of cannabis during adolescence directly might be associated with robust immunomodulatory effects and altering cytokine levels, inducing apoptosis in lymphoid cells, and stimulating the production of immune suppressor cells and consequently weakening immune defenses posing individual vulnerable to certain pathological conditions. In addition to the behavioral and neurobiological changes, the emerging evidence suggests that cannabis use during pregnancy also elicits epigenetic changes with an impactful change in neurodevelopmental, attention deficit, risk of brain damage, impaired memory, and decreased growth. Although changes in certain genes have been associated with addiction, epigenetic pathways and molecular mechanisms linked to cannabis use during pregnancy are not well defined. The changes in the epigenetic process with the use of cannabis during pregnancy are limited in comparison to adulthood. However, if there are genetic changes with the use of cannabis during pregnancy that are associated with long lasting effect in adulthood and account for neurodevelopmental and behavioral changes will be essential to dissect out in future studies. The concept of low health risk with the use of cannabis is changing, and growing evidence from epidemiological and clinical observations argue in favor of neurobiological, neurochemical, and behavioral changes, including psychosis, anxiety, depression as well as neurological diseases that are associated with cannabis use. Most importantly, the use of cannabis during pregnancy and adolescence posed these two highly vulnerable stages to well-connected events, including oxidative stress, inflammation, loss of immunity, damage to cell organelles, and excitotoxicity, critical determinants of progression and deterioration of neuronal cells and neurological diseases as discussed as a main thrust in the proceeding section of this review.

## 17. Aging, Brain Development, and Cannabinoid Receptors

Aging is the process that is associated with gradual damage at the cellular and molecular levels over time and leading to loss of physiological integrity as well as functional impairment, and it is a risk factor for most if not all human pathological conditions and all living organisms. The progressive changes in biological systems, susceptibility, and vulnerability to environmental changes, as well as certain pathological conditions often integrated with individual aging in a distinct manner. Aging is a critical determinant of impaired cognitive function and gradual memory loss not only in disease states but also in the healthy population. Aging with progression and deterioration of neurodegenerative diseases is one of the well-established risk factors in most neurological diseases. Certain key pathological features seen frequently during aging are often observed in most, if not all, neurological diseases. The commonality, including Ca^2+^ homeostasis, perturbed mitochondrial function, and oxidative stress, exists between aging and neurological diseases [205]. Although the causative factors for aging are not well understood, previous studies believed that immune response might play a major role. Several key events, including proliferation, survival, differentiation, and migration involved in the process of development, are modulated with the use of cannabinoids in addition to the significant changes in neurotransmitters maturation [270,271]. There is evidence supporting increased Ca^2+^ influx in the hippocampus, oxidative stress, and increased membrane lipid peroxidation in older population. Furthermore, increased damage due to sustained oxidative damage results in apoptosis [272,273,274,275]. Previous studies have also demonstrated the release of cytokines, as well as induction of lipopolysaccharide (LPS), and the induced release of cytokines including interleukin 6 (IL6) and interleukin 1B (IL1B) in the aging brain [276]. Together, it is conceived that neurodegeneration and chronically enhanced inflammation might play an important role in brain aging [277]. Although this is not the aim of the present review, the role of cannabinoids in aging and developmental changes in the brain can be best explained with the use of cannabinoids in perinatal and adolescence periods. Several previous studies have shown a detrimental role of eCBs and CBR subtypes in events including cell proliferation, differentiation, and migration involving the developing process of the brain. It should also be noted that the use of cannabis during pregnancy and adolescence is associated with behavioral and neurological effect that may end up in long-lasting not only neuropsychiatric disorders but also neurological diseases as well. No doubt different chemicals extracted from the cannabis plant may have different type of effects on developing brain. The effect can be worse in teenagers using new cannabis, which are several times stronger than the cannabis used in the past. Moreover, freely available cannabis due to legalization in many countries and the increasing trend of cannabis use in the young generation is posing challenges not only to the developing brain but to society, families, and the healthcare system as well.

In this context, the role of ECS in aging is not conclusive; however, studies support the cognitive deficit in the lack of CB1R [278,279]. In the postnatal brain, eCB through CB1R are associated with regulating synaptic plasticity at glutamatergic neurons in the cortex [280,281,282]. Previous studies have shown reduced CB1R-positive neurons and mRNA in a region-specific manner [283,284]. The cognitive deficit in the aged brain associated with changes in neuroinflammation probably implicates eCB-mediated regulation of glial activity. Such supposition was further supported by observation demonstrating the loss of spatial learning and neuronal loss in the hippocampus in mice lacking the CB1R gene (Cnr1 gene) [277]. Albayram Onder et al., further emphasized that the loss in hippocampal neurons is not associated with neurogenesis or epileptic seizure [277]. In parallel to these observations, the hippocampus in the absence of CB1R, exhibited augmented inflammation, increased numbers of astrocytes, activated microglia, and a high level of cytokines expression. Furthermore, the loss of pyramidal neurons and inflammation, while exacerbating each other, are also believed to promote cognitive deficit in a concert [277]. Like CB1R KO animals, deletion of CB1R on GABAergic neurons resulted in increased inflammation in the hippocampus, which was not seen upon CB1R deletion on glutamatergic neurons [277]. Altogether, these results suggest a neuroprotective role of CB1R activity on GABAergic neurons in the hippocampus in age-dependent cognitive deficit, which is associated with decreased inflammation and protection of pyramidal neurons [277].

## 18. Cannabinoids and Neurodegenerative Diseases: What Is the Link?

**Figure 8:**
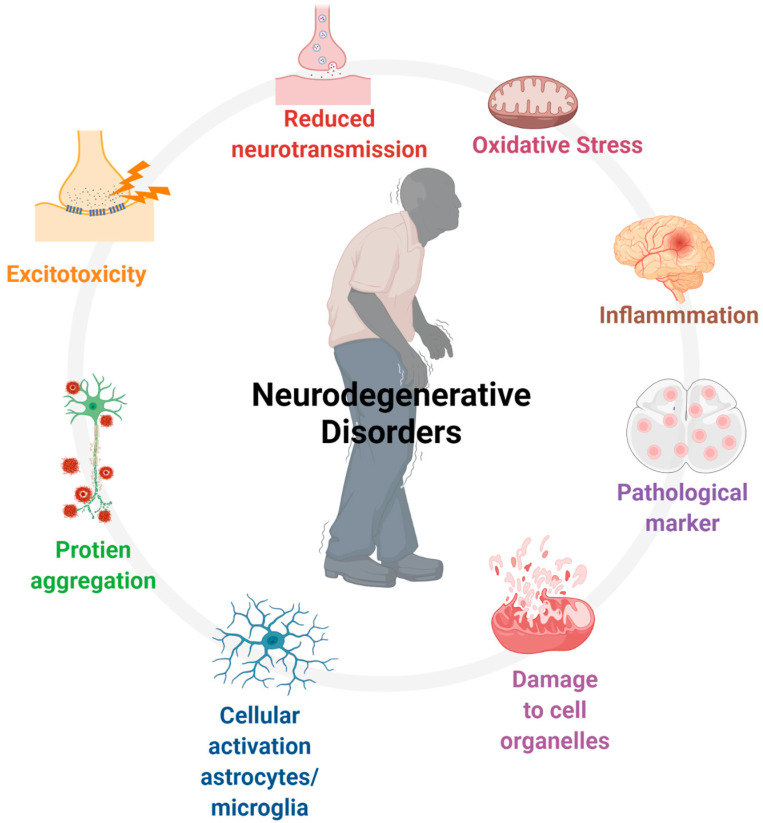
Representative illustration displaying several pathological events that might play crucial role in gradual progression of neurodegeneration diseases. This figure was created with BioRender.com.

Most neurological diseases are associated with or well recognized by specific pathological hallmarks and genetic molecules. However, the process of gradual neuronal loss in most neurological diseases is linked with well-articulated events, which exert a crucial role in disease progression (Figure 8), including neuroinflammation, oxidative stress, or generation of reactive oxygen species (ROS), excitotoxicity, and impaired adult neurogenesis, which consequently leads to damage to the cell organelles, i.e., mitochondria and perturbed energy metabolism.

Accordingly, prior to addressing the role of cannabinoids in neurological diseases, the contribution of cannabinoids is discussed in these detrimental conditions linked to neuronal loss in multiple neurodegenerative conditions. Several previous in vitro and in vivo studies support the ability of cannabinoids to suppress inflammation, oxidative stress, and excitotoxicity, as well as improve cell viability. Having seen no side effects and lack of psychotic effect, CBD is a well-approved drug by Health Canada for the treatment of pain, inflammation, and MS. Although several earlier studies support the protective role of CBD in many neurological or peripheral diseases involving multiple mechanisms, the inhibition of inflammation, oxidative stress, and excitotoxicity can be considered the prominent mechanism for the beneficial role of CBD. Furthermore, the role of cannabinoids, specifically through the activation of CB2R, in suppressing aggregated proteins, which are associated with many neurological diseases, has been proposed [285]. In addition, the newly emerging concept regarding neurogenesis and the role of cannabinoids is also discussed in reference to neurological diseases. Despite its significant neuroprotective role, the cannabinoids can also exacerbate these disease conditions. Next, certain conditions that promote neurodegeneration and are possibly blocked by cannabinoids are discussed in support of the role of cannabinoids in neurodegenerative diseases.

### 18.1. Genetic Variation in Endocannabinoid System and Neurological and Psychiatric Disorders

The interaction between genetic changes and environmental factors in the progression of neurological diseases and neurophysiological or psychiatric disorders is well established. A vast variety of functions in CNS are mediated by CB1R and CB2R, which are encoded by the CNR1 and CNR2 gene, respectively. The functional implications of genetic variance of ECS as a risk factor of neurological diseases and psychiatric disorders in humans and animals are well understood and growing evidence supports the crucial role of genetic variation in ECS in the failure of treatment of neurological diseases and neurophysiological disorders. It is believed that the genetic composition of psychiatric disorders is more complex and heterogeneous and has been extensively studied than neurological diseases. Studies have also demonstrated the impact of genetic variance on receptor expression with deleterious effects with cannabis use. Here, the impact of genetic variation in ECS is briefly discussed on neurological and psychiatric diseases. The dysregulation of endocannabinoids either at the level of protein or gene has been implicated in pathogenesis of several neurological diseases including AD, PD, HD, MS, and ALS as well as psychiatric disorder such as schizophrenia, depression, stress, and anxiety. Furthermore, genetic variation in ECS also impact inflammation, ROS, excitotoxicity, and neurogenesis, crucial factors contributing the worsening of most neurological diseases.

Anxiety disorder and symptoms have been associated with genetic variance selectively with CB1 but not with FAAH. Previous studies carried out in an animal model with a genetic disruption in FAAH show enhanced AEA-mediated signaling resulting in anxiolytic effect, whereas disruption in CB1R has been associated with an anxiogenic and depressive-like effect by modulation of ECS signaling [286,287,288]. Furthermore, a single nucleotide polymorphism in CNR1 is not only associated with depression but also interferes in treatment [289]. Although controversial, genetic variance might reduce levels of CNR1 that interact with cannabis use and associated with abnormal connectivity and behavior during working memory. Although CB2R is relatively expressed less in brain than CB1R, it is worth mentioning here that a polymorphism in the CNR2 gene encoding CB2R is linked to schizophrenia and is associated more efficiently with depression in the Japanese population [290]. It should also be noted that rare genetic variations in ECS, including the genes CNR1 and CNR2 encoding CB1R and CB2R, respectively, as well as the genes for the degrading enzymes DAGLA, MGLL, and FAAH have been reported in patients with neurological disorders [291]. Multiple genetic factors are involved in pathogenesis of MDD; one of them is genetic variance in retrograde eCB pathways.

Hillard et al., demonstrated that genetic polymorphisms in CB1R and FAAH exhibit a crucial role both in normal and several pathophysiological conditions [253]. Authors further emphasized that disrupted genes for CB1R and FAAH are associated with symptoms and diagnosis of several behavioral abnormalities and substance use disorders. While the functional significance of genetic variation in FAAH is well understood, the functional significance of changes in CNR1 is largely elusive. As mentioned above, genetic variations in ECS are crucial in neuropsychiatric disorders. To support this presumption, growing evidence has described that in human patients, a polymorphism in CNR1 and CNR2 is associated with schizophrenia, autism, depression, anxiety, bipolar disorder, and depressive syndrome [292,293,294,295,296]. In addition, human patients also display polymorphisms in DAGL-alpha that is linked to focal cortical dysplasia [297]. Studies also support gain of function with polymorphism in FAAH that is linked to lower anxiety via involving amygdala. In addition to the major enzyme FAAH1, which hydrolyzes AEA in mammals, recent studies have identified FAAH2, which is absent in rodents including rat and mouse but present in human [298]. Whether defects in FAAH2 activity is associated with psychiatric/neurologic phenotype in lack of sufficient data is not well established yet. There are conflicting observations regarding the distributional pattern of FAAH2 in human brain [298,299]. Since mouse and rat are devoid of FAAH2, in such cases any comparable observation between functional significance and implication of FAAH2 absence in human with mice knock out with FAAH1 should be interpreted carefully. Although controversies exist due to lack of sufficient observation on FAAH2 but FAAH seems to be a potential therapeutic intervention in neurological diseases and psychiatric disorders.

In addition to the direct changes on disease progression, the impact of genetic variation on brain development, cognitive and memory function, synaptic transmission, influence on neurotransmitter imbalance due to affected inhibitory and excitatory input and different signaling pathways might involve indirectly in pathogenesis of neurological diseases. Previous studies have shown DAGLA variant on neurological function and development through the regulation of 2-AG and ECs synthesis. Previous studies have also shown association between single nucleotide polymorphism and memory processing [300,301]. As discussed above, the implication of functional consequences of genetic variation in ECs in addition to neurological diseases and psychiatric abnormalities might also play significant role in brain development and aging with a determinant effect in progression of neurodegeneration. Therefore, taken together existing evidence suggests that changes in endocannabinoids system at the molecular level may serve as a crucial contributing factor in pathophysiology of neurological diseases.

### 18.2. Cannabinoids and Regulation of Inflammation

Growing evidence in individuals with psychotic abnormalities supports the changes in the inflammatory system [302,303,304]. Changes in the immune system in response to injuries in CNS have been associated with multiple pathological conditions. Moreover, a close association between cannabis use and a high risk of psychosis is often seen. Although controversial, it was interesting to correlate this with inflammation-induced cytokines, namely IL-6, in addition to the negative relationship between the two [305]. Concomitant observations from multiple studies support the concept that individuals with cannabis use display perturbed inflammatory systems and also show a high incidence of vulnerability when compared with the non-cannabis user. The presence of CB2R in immune cells and enhanced expression of CB2R in conditions like an inflammatory insult is a good excuse to propose the role of CB2R in the modulation of immune cell response [306,307]. However, the regulatory effect on immune cells function is not restricted to CB2R only; the association of CB1R has also been proposed due to changes in cytokine in response to CB1R activation [306,308,309,310,311]. This is further supported by the observations in CB2R-deficient mice lacking immune loss. The neuroprotective role of cannabinoids has also been reported in neuroinflammation that occurs during normal aging [306]. Growing evidence indicates modulation of the immune system in the presence of cannabinoids via binding to CB1 and CB2R subtypes as a potential therapeutic avenue (discussed later in detail). This is true for CB2R, in part because of the role of receptor in regulation of proinflammatory inducer cytokines. Furthermore, in an in vitro condition using hippocampal neuronal cells, the addition of 2-AG or increasing endogenous 2-AG has been neuroprotective against inflammation induced by LPS or IL-1 B, glutamate, and kainite-induced excitotoxicity, as well as amyloid-induced toxicity. 2-AG mediated neuroprotection was further supported by limiting COX2 through activation of CB1R-mediated transcription factor NF-kF signaling pathways [312,313,314]. Taken together, existing data revealed endocannabinoid-mediated inhibition of proinflammatory cytokines, and enhanced production of anti-inflammatory cytokines is associated with neuroprotection, and cannabinoids are potential therapeutic drugs in several inflammatory conditions [315].

### 18.3. Cannabinoids as Suppressant of Oxidative Stress

The role of oxidative stress in neurotoxicity is well established, and previous studies have shown that CNS is highly vulnerable to ROS, which accounts for poor antioxidant defense mechanisms and poor energy preservation of CNS. Neuroprotection by CB1R agonist WIN 55212-2 in a rat model of focal ischemia is prevented by CB1R antagonist SR 141716A, supporting the role of cannabinoids in oxidative stress [316,317]. Furthermore, some cannabinoids also afford neuroprotection against oxidative stress even in the absence of any binding to CB receptors supporting neuroprotection independent of CB1R. Although phenolic cannabinoids have direct antioxidant action due to their lipophilic nature, these drugs might exert a neuromodulatory role for certain membrane-bound neurotransmitters at the cell membrane by changing membrane fluidity [317]. Cannabinoid might be involved in the regulation of downstream signaling pathways, as shown by estradiol, which occurred independently of receptors [318]. Taking these observations into consideration, the MAP kinase signaling pathway, which is regulated in CBR-dependent and independent manners, could be a plausible mechanism for neuroprotection [207,319].

The increased expression level of endocannabinoid during brain injury and in vivo in ischemic brain damage support neuroprotection in a CB1R = dependent manner. Furthermore, suppression of glutamate-induced neurotoxicity post-CB1R activations also argues for receptor-dependent protection in conditions like oxidative stress. Giovanni et al., proposed that CB1R-dependent antioxidant neuroprotection with some compounds devoid of the phenolic group that is associated with most antioxidant effects [317]. Whether endocannabinoids added exogenously with receptor-specific agonists interfere in oxidative pathways is worth mentioning here. Although, beyond the scope of this review, the use of marijuana smoke, which contains psychoactive content, is also associated with increased oxidative stress and cell damage as well as perturbed cell function.

### 18.4. Excitotoxicity and Cannabinoids: Neuroprotection

The concept that cannabinoids might exert a beneficial effect in excitotoxicity: a mechanism by which neuronal damage occurs in most neurological diseases emerged from the inhibition of glutamate release from the presynaptic terminal following activation of CBRs. This act of CBR is sufficient enough to support the notion of cannabinoid-mediated neuroprotection in excitotoxicity. Both CB1R and CB2R subtypes have shown neuroprotection from NMDA-induced toxicity with a poorly defined mechanism. Using hippocampal neurons in culture, Shen and Thayer described neuroprotection in the presence of a CBR agonist [320]. Chiarlone et al., reported the presence of CB1R at GABAergic and glutamatergic neurons and excellently discriminated neuronal cells expressing CB1R is neuroprotective in excitotoxicity [321]. These observations revealed that the CB1 receptors present on glutamatergic neurons are associated with the neuroprotective effect of endocannabinoids. These results further extended in a model of excitotoxicity and HD (Discussed in detail under section HD and Cannabinoids receptors).

### 18.5. Neurogenesis and Cannabinoids: Role in Neurodegenerative Diseases

The formation of new neurons in the brain, referred to as adult neurogenesis, is making significant progress with specific emphasis on neurological diseases and psychiatric disorders. Neurogenesis, believed to occur during embryonic development, is also seen in the adult brain. Neurogenesis is restricted to certain brain regions and is most studied in two brain regions, including the hippocampus and olfactory bulb. However, recent studies have also described neurogenesis in additional brain regions including the cortex and hypothalamus [322]. The process of neurogenesis that is regulated by multiple factors, including neurotransmitters, trophic factors, adrenal and sex hormones, as well as inflammatory cytokines [262], is associated with the formation of new neurons as well as their integration into the existing complex communicating network in CNS. Neurogenesis plays a crucial role in neurite outgrowth and elongation essential for neuronal functionality and integrity. Perturbed neurite formation is often seen in neurological diseases attesting to the role of neurogenesis in neurological diseases [262,323]. The most studied connection between cannabis and neurogenesis emerged from ECS-mediated regulation of factors involved in neurodegenerative diseases including damage to neuronal processes, neuroinflammation, oxidative stress, neurotransmitters, and regulation of growth factors. In neurological diseases, perturbed neurite formation is linked to impaired cognitive function, and studies support the role of cannabis in neurite outgrowth and elongation. The direct evidence for the role of ECS in neurogenesis is still sparse and evidence in support of ECS role in neurogenesis emerged from CB1R deletion studies displaying perturbed neurogenesis [324]. However, growing evidence indirectly supports the crucial role of ECS in neurogenesis. The role of neuroinflammation in brain aging and the gradual progression of neurological diseases has also been reported to be associated with decreased neurogenesis. Furthermore, negative regulation of neuroinflammation by CBR subtypes in a receptor-specific manner is well established and indicates the role of cannabinoids in neurogenesis. Altogether, in CNS, a close interaction is established between ECS, the immune system and neurogenesis with a significant impact in neurodegenerative diseases [29,262,323]. Importantly, their role in proliferation of neural progenitor cells in neurogenesis attest to the role of ECS [323]. Also, activation of CB1R in the presence of arachidonyl-2-chloroethylamide (ACEA) promotes neuronal differentiation and maturation of neural stem cells [323]. In parallel to the CB1R contribution in neurogenesis, the contribution of CB2R in NSC differentiation is receptor agonist-specific [325]. An additional avenue explored for the role of cannabinoids in neurogenesis is CBR subtype-mediated regulation of BDNF, which is required for adult neurogenesis. In most neurological diseases, a decreased expression level of BDNF is observed. Whether CBR subtypes upon activation increase BDNF in neurodegenerative diseases is not well understood; however, increased levels of BDNF have been reported in response to CB1R activation [326]. Endocannabinoids, in addition to their crucial role on CBR subtypes, also play a critical role in regulation of neurogenesis and expression levels of BDNF at the cellular level [323]. Impaired neurogenesis resulted in failure of neuron replacement as well as loss in memory and cognitive function, which is associated with perturbed neurite formation and outgrowth.

Endogenous cannabinoids, 2-AG and AEA, afford some beneficial effects in neurodegenerative conditions as well as in neuroinflammation. In β-amyloid (Aβ)-induced neurotoxicity, the role of AEA and CBD has been proposed as an inducer of neurogenesis [327]. In neurological diseases and psychiatric disorders, neuronal loss, interrupted neuronal communication, and perturbed memory and cognitive function are frequent observations. Growing evidence support impaired neurogenesis in neurological diseases, including AD and HD [262,328,329]. In HD cases, neuroprotection is often seen with the use of cannabinoids, specifically with the use of THC; however, loss in the expression level of CB1R and unexpected side effects warn the use for clinical purposes. Cannabinoids have shown significant diversity in regulating adult neurogenesis in a drug-, dose-, and time-dependent manners [330]. Recent emerging studies on neurogenesis support the role of cannabinoids through activation of CB1R and CB2R involved in the regulation of embryonic and adult CNS. Moreover, studies also emphasized the role of cannabinoids in neurological diseases and psychiatric disorders and have been reviewed in detail elsewhere [331].

## 19. Cannabinoids and Perception of Pain

Pain is a serious and growing global problem among adults with no universally effective treatment. At present, 1.5 billion people suffer from pain. Multiple factors, including immune deficiency, neurodegenerative diseases, metabolic diseases, and many other environmental factors, might contribute to pain initiation. The analgesic property of cannabinoids in the experimental model of hyperalgesia supports the role of cannabis in pain relief, specifically in inflammation-induced pain as well as neuropathic pain. The expression of CBRs in central and peripheral regions, specifically in brain areas that are involved in pain, including the spinal cord thalamus and amygdala, associated in pain perception and nociceptive input provides the first indication for the role of CBR subtypes [50,332]. The use of endogenous cannabinoids in pain relief has been proposed through various mechanisms, which are comparable in some ways and different in others from opiate use [333]. The existing results collected from preclinical or clinical studies support the role of cannabis in pain and inflammation. Amongst the most clinical use of cannabis, pain relief is the prominent therapeutic use of cannabis, including Δ^9^-THC and CBD. Upon activation, CBR subtypes inhibit the release of inflammation-inducing molecules, modulate the nociceptive threshold, and enhance the analgesic effects of endogenous opioids [50,332]. Recently, the availability of selective antagonists of CBRs, generation of receptor subtypes knockout and transgenic mice, as well as the presence of eCB have significantly extended our knowledge about cannabis. The neuronal cells confined in dorsal root ganglion synthesize CB1R and also display colocalization with vanilloid receptors, which use Anandamide as an effective agonist [334].

Although endogenous opioids are known to inhibit pain initiation through binding to their cognate receptors, namely opioid receptors (OPRs), emerging observations also emphasized the crucial role of eCB in this direction. Whether the role of eCB in the regulation of nociception signal in the peripheral system is comparable to CNS is largely elusive. The inhibition of pain-associated behaviors following activation of CB1 and CB2 receptors confined to the peripheral system has been shown to function in a comparable manner as normally seen with the use of opioids [335,336,337,338,339]. Furthermore, the ablation of CB1R in primary nociceptive neurons leads to worsening pain-associated behaviors [340]. Previous studies have also described the increased levels of AEA at the peripheral site with certain clinical conditions, including neuropathic pain and inflammation [341,342]. In addition to AEA, previous studies also support the role of 2-AG in the regulation of nociception signals in the peripheral system [340,343].

The analgesic effect of CB2R activation in conditions like inflammation and neuropathic and bone cancer pain is normally seen. Although, the presence of CB2R in CNS is well accepted, the upregulation of CB2R at the site, which is involved in the process of nociception, has been reported [344,345,346]. However, important observations regarding the CB2R, like immunoreactivity in dorsal root ganglion, are an opportunity to delineate the molecular mechanism for the role of CB2R in antinociceptive. The inhibition of inflammatory and neuropathic pain is seen following the activation of CB1R and CB2R subtypes. However, the use of most drugs that work through activation of CB1R is very limited due to unwanted side effects. Despite some limitations with the use of cannabinoid in pain relief, Δ^9^-THC alone and in combination with CBD is well placed as a choice for the relief of neuropathic pain in MS patients in Canada. Moreover, suppression of inflammatory and neuropathic pain has been reported with the use of CBD alone [347]. At present, a large number of countries, including Canada, authorize the use of Nabiximols, a cannabinoid combination of Δ^9^-THC and CBD for pain. Whether cannabis can be used as an effective and efficacious alternative to opioid for the treatment of acute and chronic pain or may help to understand complex neurobiology involving in pain is not known.

## 20. Therapeutic Implications of Cannabinoids in Neurological Diseases

Neurological diseases represent the most complex and heterogenous pathology and are associated with multiple factors including aging, excitotoxicity oxidative stress, neuroinflammation, gliosis, changes in different neurotransmitters, augmented Ca^2+^, mitochondrial damage and importantly gradual loss of memory and cognitive function and neuronal loss in addition to aggregation and mutation of certain proteins in disease-specific manner. The clinical use of cannabinoid-based drugs is a question of debate. Previous studies have shown the beneficial role of cannabinoids in appetite, pain, nausea, sleep and psychosis as well as key factors discussed above. A number of evidence in support of the neuroprotective role of cannabinoids exist including anti-proliferative effect via regulation of multiple downstream signaling pathways, anti-angiogenesis through regulation of vascular endothelial growth factor (VEGF) and nerve growth factor (NGF) and anti-inflammatory effect via modulation of cytokines release and activation, blocking the oxidative stress and mitochondrial damage as well as activation of NMDA receptors, promotion of neurogenesis, inhibition of N and P/Q type voltage-dependent Ca^2+^ channels and activation of inwardly recertifying K channel. These interconnected events play a determinant role in the process of how neurons die in most neurodegenerative diseases. Recent studies have begun to address the role of ECS in neurological diseases. Neurodegenerative diseases have drawn significant attention for the use of cannabinoids and have been a potential target in developing an effective therapeutic drug. Previous studies from different experimental conditions, including psychiatric disorders and neurological diseases, have shown changes in eCB levels in plasma and cerebrospinal fluid (CSF) in AD, PD, HD, epilepsy, depression, MS, migraine headache, anxiety and stress as well as in schizophrenia [140,348,349,350,351,352,353,354,355,356,357,358,359,360]. Despite significant advantages from using cannabinoids in the above pathological conditions, cannabis use is still controversial. Furthermore, conclusive observations, lack of data and molecular determinants supporting the knowledge gap, and assisting in constructing an appropriate clinical guideline implicating cannabis use in clinical practice are largely awaited. In addition to the beneficial effects, studies also support adverse effects with the use of cannabinoids. At present there is no effective treatment for most neurological diseases. This section of the present review aims to describe the latest findings and discuss the contribution of ECS via the activation of two cell surface receptor proteins, namely CB1R and CB2R, in neurodegenerative diseases and psychiatric disorders.

## 21. Epilepsy and Cannabinoids

Epilepsy is the most common, complex, and devastating chronic disorder of CNS, making people vulnerable to recurrent seizures. There are several factors including genetic syndrome, brain injuries, infection, and stroke, which have a unique characteristic feature of complicity. Studies support that epilepsy can affect people of all ages; however, growing evidence argues that epilepsy most commonly affects children and the elderly. Also, cognitive, psychological, sensorimotor, and social consequences are also associated with epilepsy. Epilepsy is one of the neurological abnormalities which draws the first attention of cannabinoids as an effective treatment option. Neuronal excitability that leads to enhanced seizure activity is well connected with inflammation, excitotoxicity, and ROS, and the inhibition of neuronal hyperexcitability with the use of cannabinoids is the possible mechanism. The historical record of the anti-epileptic effects of the CB1R dates back centuries [361]. Case reports on the beneficial effects of cannabinoids on epileptic patients became available only after the identification of Δ^9^-THC [362,363].

Some positive results have been reported in children with the use of Δ^9^-THC. However, studies also suggest increased seizure frequency after marijuana smoking [364]. This paradoxical effect of cannabinoids on epilepsy is not only seen in human studies but has also been reported in animal models [365,366]. The activation of the CB1R by AEA has been shown to inhibit electroshock-induced seizures in rats [366]. Conversely, CB1R activation in FAAH knockout mice displays increased susceptibility to kainic acid-induced seizures [365]. The reduced level of AEA in CSF from temporal lobe epilepsy with no changes in 2-AG describes specific and selective changes in eCB. Interestingly, hippocampal and cortical tissue samples collected from patients with temporal lobe epilepsy exhibited relatively high levels of AEA than 2-AG. The alteration of the endocannabinoid system following epilepsy is cell type-specific. This concept is supported by previous animal studies showing that CB1R retrograde signaling is selectively enhanced at inhibitory but not at excitatory synapses, resulting in a persistent potentiation of DSI but not DSE in febrile seizures, which leads to hyper-excitability of neurons, thus, contributing to the exacerbation of seizures [367,368]. Moreover, this CB1R-mediated enhanced suppression of inhibitory neurons is phase-dependent as well. Hippocampal tissues from epileptic patients in the acute phase of epilepsy display decreased CB1R density, especially in the dentate gyrus, whereas in patients in the chronic phase of epilepsy, an upregulation of CB1R has been observed [369,370,371,372]. Previous studies using different experimental models of seizures have described the anticonvulsant effect of CBD. In brain regions, including the cortex, striatum, and hippocampus, GABA-releasing terminals exhibited higher expression of CBR when compared to glutamatergic. This might play a role in the bidirectional effect of Δ^9^ THC, which further emphasizes the dose-dependent pro and anti-convulsive effects of Δ^9^-THC in a dose-dependent manner. Moreover, the use of cannabinoids as orphan drugs for the treatment of epilepsy is approved by the Food and Drugs Administration [373]. The use of CBD, one of the most potent non-psychoactive cannabinoids, is a well-recognized drug for seizure management in children with Lennox–Gastaut and Dravet syndrome with no side effects [374,375,376]. Furthermore, synthetic cannabinoids have not been successful, rather, they produce pro-convulsive effects through the activation of transient receptor potential vanilloid (TRVP) channels [377,378]. The mechanism of action of CBD, in addition to CB1R and CB2R, also involves adenosine, GPR55, and serotonin receptors, as well as TRVP channel activation and inhibition of T-type voltage-gated calcium channels [21,78,379].

## 22. Alzheimer’s Disease and Cannabinoid Receptors

AD is the most common and prevalent age-dependent progressive and irreversible neurological disease that is characterized by the deposit of Aβ, accumulation of senile plaques (SP), and formation of neurofilament tangles (NFT) and the hyperphosphorylated microtubule associated protein tau in the brain as a histopathological hallmark of the disease [380]. The increasing number of patients with AD globally in part are associated with extended life span and lack of effective treatment to interfere with the prominent causes of disease, including neuronal loss and deterioration of cognitive function and memory loss. AD represents complex and heterogeneous pathology at the levels of morphology, biochemistry, and molecular biology that consequently leads to the loss of multiple brain functions. The mutation/changes in amyloid precursor protein (APP) and presenilin (PS), as well as phosphorylation of tau protein, are the prominent causes of disease [381]. In addition, multiple other factors associated with the gradual progression and severity of disease include excitotoxicity, increased calcium, oxidative stress, cell organelles damage and failure, inflammation, perturbed neovascularization, impaired cognitive function, memory loss and neuronal cell death [380,382,383]. In the search of effective treatment, drugs targeting accumulated Aβ and hyperphosphorylated tau are failed, whereas drugs working on cholinergic and NMDA receptor are only improving disease symptoms. At present, due to these multiple factors, there is no effective treatment, but there are some options to slow down the progression of the disease and extend the life span. Amongst such treatment opportunities, cannabinoids are making their place and emerging as one of the important drugs in the treatment of several neurodegenerative diseases beyond the pain perception and appetite stimulator in cancer patients. Cannabinoids interfere in most of the events involved in neurodegeneration, as discussed above, and afford neuroprotection as the most effective therapeutic intervention in AD [384]. The use of natural and synthetic cannabinoids through activation of CBR subtypes in a different model of AD exhibited beneficial effects, including suppression of Aβ induced toxicity, tau phosphorylation, and also involvement in the intrinsic defense mechanism of the brain [327]. An increasing number of studies support and implement cannabinoids in the pathogenesis of AD with several neuroprotective properties. It is interesting to note that during the asymptomatic stage of disease, multiple factors, as discussed above associated with progressive neuronal damage, are modulated in the presence of endocannabinoids and support the beneficial role of cannabinoids in AD. Maresz et al., described improved cognitive function and suppressed inflammatory response with the use of CB1R agonist ACEA in the AD transgenic model [264].

The concept that neuroinflammation is associated with AD progression and neuronal cells damage due to microglial activation prompted the notion that the suppression of proinflammatory microglial cells may also account for the neuroprotective role of CB2R in AD. The use of CB2R agonist blocks the production of proinflammatory cytokines in Aβ administered rat brains, whereas transgenic mice displayed suppressed number of activated microglial cells and cytokine levels [327,385,386,387,388]. Moreover, in view of previous studies and recent interest in preventing microglial activation and blocking inflammatory inducers, i.e., cytokines might serve as a potential therapeutic treatment for AD. Grunblatt E et al., showed the expression of CB2R mRNA in blood samples from AD patients and associated with impaired cognitive function [389]. These results were further extended by the observations displaying an increased expression of EC degrading enzyme FAAH in AD [390]. Earlier studies have also described the expression of CB2R in plaques formation in the brain region associated with AD and increased receptor expression in the advanced stage of the disease. Direct association of CB2R emerged from inhibiting of Aβ mediated activation of microglia following icv administration of cannabinoids in an experimental model of AD. CB2R agonist has been shown to inhibit Aβ induced neuroinflammation and also exhibit Aβ clearance and improved cell viability [279,281]. Furthermore, the chronic administration of CB1R and CB2R agonists in combination plays a role in memory function in rats and mice [267,281,282]. In a genetic model of tauopathy, Sativex, a synthetic combination of Δ^9^-THC and CBD, afford the blockade of microglial reactivity with no obvious implication of CB2R suggesting CB1R anti-inflammatory role [391]. CBD is devoid of binding affinity to both CBR subtypes but exerts an anti-inflammatory effect in AD; in such conditions, the implication of PPARγ was proposed for the neuroprotective properties of CBD in AD [392].

### 22.1. Neurotransmitters Modulation and AD: Role of Cannabinoids

The perturbed synaptic transmission in AD resulted in decline of cognitive function, with the notion that the retrograde messenger system exerts a crucial role in synaptic efficacy and neuronal activity by regulating the release of the neurotransmitters in a precise manner. It is plausible to predict the role of cannabinoids in the pathogenesis of AD. Mulder et al., described the correlation between AD and ECS [393]. In the hippocampus of AD patients, at the levels of protein, no alterations in CB1R expression were observed when compared to age-matched control but exhibited significant increase in sn-1-diacylglycerol lipase a and b isoforms, synthesizing 2-arachidonoyl glycerol at an advanced stage of the disease. The authors defined the significance of ECS in the formation of senile plaques, microglia, and formation of NFTs. In another set of studies using mouse hippocampi superfused with Aβ resulted in changes in 2AG-regulated signaling, which leads to the sustained depolarization-induced suppression of inhibition that possibly contributes to synapse silencing in AD brain. In AD, enhanced eCBs signaling, specifically in the vicinity of senile plaques, has been associated with synaptic failure. Δ^9^-THC -mediated inhibition of acetylcholinesterase (AChE) resulted in an increased level of acetylcholine (ACh) and blocked Aβ aggregation that implicated Δ^9^-THC binding to the specific site of AChE involved in amyloidogenesis [394]. Furthermore, like AChE inhibitors, cannabinoids are also associated with the suppression of Ca^2+^ and excitotoxicity.

### 22.2. Changes in Endogenous Cannabinoid and Cannabinoid Receptors in AD Brain

The changes in CBRs in the AD brain and their role in the pathogenesis of AD are most controversial or indistinct. In contrast to no changes in the cortex, decreased expression of CB1R in the hippocampus has been described in the AD brain [140,259,393,395,396,397,398]. Growing evidence from the experimental model of AD and autopsy brain tissues attest to the role of ECs in the modulation of cellular and biochemical events associated with neuropathogenesis and progression of AD. The changes in the distributional pattern of CB1R also revealed region-specific expression in microglial and neuronal cells of the hippocampus, displaying strong CB1R in microglia in the association of plaques formation and loss of receptor expression in neuronal cells [267,268]. Moreover, no functional relation is established between the expression of CB1R and deteriorated cognitive function in AD. It should also be noted that using CB1R KO animals, loss of learning and memory in an age-related manner have been reported, as well as with the use of Phyto-CBs, the role of EC signaling is supported in the aging brain [278,399]. In contrary to CB1R, increased expression of CB2R has been reported in microglia and senile plaques [140,396,397]. Furthermore, such correlation exists between CB2R, Aβ and plaque formation but not with cognitive function. The perturbed relation between CBR and its effector in AD is due to nitrosylation of the receptor [393,396]. Mulder et al., proposed that suppressed presynaptic, as well as postsynaptic 2-AG degradation in parallel to increased and ectopic 2-AG synthesis, might be involved in the disruption of retrograde signal and, as a result, intensify or worsen synapse damage in AD [393].

Recently, ECS has drawn significant attention to understanding the molecular mechanism for their role in the pathogenesis of AD and possible therapeutic alternatives. ECs are becoming a potential therapeutic alternative approach in AD treatment. In AD, EC plays two different types of roles depending on receptors subtypes involved. Increased EC via activation of CB2R inhibits proinflammatory microglia and blocks the release of cytokines [400]. In contrast, CB1R can protect neuronal cells from Aβ induced neurotoxicity as well as from Aβ induced amnesia in the hippocampus to involve in learning [401,402]. Furthermore, depending on the severity of AD, CB1R at an early stage of neurodegeneration improves cognitive function by inhibition of apoptosis and gliosis, whereas at a later stage of the diseases, it promotes Aβ induced loss probably by blocking residual activity as suggested previously [360]. It is uncertain whether CB1R is linked with synaptic plasticity in AD. In addition, studies support the increased production of ECs in response to neuronal damage that might also function as a possible way to protect neurons from toxicity [51,403]. The alternative mechanism might involve cannabis-mediated neuroprotection in AD stemming out from HU-211 induced protection in excitotoxicity, including the inhibition of glutamate release, voltage-dependent calcium channel, and Ca^2+^ release. Although, large numbers of accumulated results are driven from animal model studies and with limited clinical observations future studies are warranted. However, suppression of neuroinflammation, improved cognitive function and memory loss, as well as the acceleration of microglial migration are some effective therapeutic considerations with the use of cannabidiol.

## 23. Huntington’s Disease and Cannabinoid Receptors

HD is a progressive dominant inherited neurological disease that is caused by extended CAG repeats in the huntingtin gene; this expansion of the gene is linked to the elongated glutamine repeats at the NH2 terminus of huntingtin protein (*Htt*). HTT is widely distributed in CNS and peripheral tissues, but still, what role the protein plays in the target tissue is not well understood. Mutated HTT results in aggregated protein through multiple processes and results in severe neuronal loss and striatal atrophy. In HD, medium-sized spiny GABAergic neurons are affected the most. As the disease progressed, neuronal loss also occurred in other brain regions, including the cortex. In contrast, a small population of medium-sized aspiny neurons expressing somatostatin (SST), neuropeptide Y (NPY), and brain nitric oxide synthase (bNOS) are selectively spared. The molecular determinants associated with demised neuronal cells are multiple, including increased Ca^2+^, oxidative stress, overactivation of NMDA receptor (excitotoxicity) probably due to a decrease in glutamate uptake and impaired glutamate transporter in HD [404], and inflammation due to activation of microglial cells and cytokines release [405]. Furthermore, mutated HTT’s ability to bind mitochondria resulted in mitochondrial damage and perturbed metabolic activity and resulted in the enhanced proapoptotic pathway [406]. In addition, the loss of trophic factor, i.e., BDNF, is also believed to cause neuronal death in HD. Such a gradual neuronal loss and disease severity develop symptoms like impaired cognitive function, motor dysfunction as well as psychological disturbances.

In context to ECS and HD, earlier studies have shown changes in ECS with gradual progression of HD. Previous studies have demonstrated the loss of CB1R in the striatum (caudate, putamen and globus pallidus) as the disease progressed. Further to the loss of CB1R, the damage has also been shown to medium spiny neurons (MSNs) and interneurons expressing SST/NPY and bNOS [407]. However, selective preservation of medium-sized aspiny neurons expressing SST/NPY and bNOS in HD is well established, but the loss of CB1R in these neurons raises a question of whether loss of CB1R is the cause of deterioration of interneurons as the disease progresses. In contrast, increased expression of CB1R in MSNs has also been reported to improve the survival of excitatory projection neurons without any improvement in motor performances of R6/2 mice [408]. A descriptive role of ECS in HD emerged from a study using HD tg R6/2, R6/1 and YAK mice and mice devoid of CB1R. Furthermore, changes in disrupted CB1R gene expression due to mutated HTT resulted in a decreased density of CB1R in HD [409,410]. HD symptoms are aggravated in the absence of CB1R, whereas the use of Δ^9^-THC resulted in improved HD symptoms, supporting the positive role of CB1R in HD pathogenesis [409]. In HD, suppressed CB1R is seen in the striatum at an early stage of the disease, whereas the occurrence of psychotic effect post-CB1R activation limited the use of CB1R in HD. It should also be noted that recent studies have drawn significant attention to the role of CB2R in the pathogenesis of HD. CB2R is primarily a receptor that is highly expressed in microglia in comparison to neurons. Palazuelos et al., reported increased microglial expression of CB2R in the striatum of HD tg mice and HD patients and proposed that CB2R might serve as a neuroprotective because in HD tg mice lacking CB2R might lead to the activation of microglia [411]. Further evidence in support for the role of CB2R in HD emerged from CB2R knock-out mice, which increases quin-induced neuronal damage in the striatum, whereas CB2R agonists are proposed to protect striatal neurons and result in suppression of motor symptoms and negative regulation of neuroinflammation [411]. Previous studies support the notion that CBR subtype activation and inhibition of the enzyme involved in the degradation of eCBs might afford neuroprotection in HD [412]. However, in parallel to these observations, rodents devoid of eCB synthesizing enzyme monoacylglycerol lipase resulted in the protection of MSN and improved motor activities in huntingtin-induced damage [413]. Nadal et al., reported tetrahydro-cannabinolic acid-mediated neuroprotection in vitro using mutated cells expressing huntingtin and other cells with mHtt-q94 expression and proposed that neuroprotection is mediated by agonism at PPARγ [414]. Consistent with these observations, we recently used mHtt knock-in (STHdhQ111/111) and wild-type (STHdhQ7/7) striatal cells and observed higher susceptibility of STHdhQ111/111 cells in response to QUIN with suppressed cell survival signaling. SST and CB1 receptor-specific agonists afford protection of cells from QUIN-induced toxicity and activated ERK1/2 in both STHdh cells. However, co-activation of Somatostatin receptor subtypes and CB1R resulted in diminished protective effects, delayed ERK1/2 phosphorylation, and altered receptor complex composition, with pronounced effects in STHdhQ111/111 cells than STHdhQ7/7 cells [121,122,415]. Vast varieties of changes in ECS have been reported in the striatum of HD, including a decreased reduction in AEA, 2-AG, and their synthesizing enzymes [416,417]. The same study also described reduced levels of 2-AG in parallel to increased levels of AEA in the cortex while hydrolytic enzymes MGL and FAAH exhibit decreased and increased levels in HD, respectively [416,417]. Cannabinoids in combination have shown improvement in symptoms associated with HD; however, such beneficial effects can only be obtained with the appropriate combination [265].

## 24. Parkinson’s Disease and Cannabinoid Receptors

Parkinson’s disease (PD), is the second most common progressive neurodegenerative disease and targets adults, affecting 1% population around the age of over 50 years. Although aging is critical factor for PD; however, symptoms of this idiopathic disease can progressively change from motor defects such as tremors to impairment of thinking and behavior. PD is characterized by the loss of dopaminergic neurons in the midbrain region, namely substantia nigra specifically in substantia nigra pars compacta, as well as loss of dopamine metabolites in the brain in addition to the accumulation of α-synuclein. substantia nigra, which consists of more than 90% tyrosine hydroxylase (TH)-positive neurons, sends dopaminergic projection to the striatum [418]. The disrupted dopaminergic circuit in the striatum resulted in bradykinesia, rigidity, and tremors are known classical features of progressive PD. In addition to the loss of dopaminergic neurons, protein aggregation, mitochondrial damage, oxidative stress, loss of antioxidant enzymes, activation of NMDA receptors and inflammation are also believed to be determinant factors in PD progression. The formation of Lewy bodies, which contain neurofilaments and aggregated alpha-synuclein protein, is also frequently seen in PD. In addition to the behavioral, biochemical, and well-established loss of dopaminergic neurons, genetic mutation has also been reported as a causative factor of PD. Earlier studies using PET analysis and autopsy tissue examination revealed increased microglial cells and proinflammatory molecules, including cytokines [262,419,420,421]. The modulation of GABAergic and glutamatergic transmission in the basal ganglia following activation of CBRs is critical in the association of ECS and PD, which support functional interaction and its effect on motor activity [422,423]. The growing evidence, including changes in cannabinoids, mediated signaling, presence of CBR subtypes in affected brain regions, and changes in ECS in basal ganglia following L-DOPA treatment, support the role of cannabis in the pathogenesis of PD [424,425]. First, relatively high expression of CB1R and levels of ECS in brain regions involve in the movement including substantia nigra and globus pallidus second, inhibition of neuroinflammation and oxidative stress are compelling evidence in support of the neuroprotective role of cannabis in PD. These observations further emphasized increasing interest in cannabinoid use in PD with a specific role in motor disorders. Importantly, following the depletion of dopamine due to the loss of TH activity in PD, ECS displayed significant neurochemical, physiological, and morphological changes. This loss of dopamine further results in enhanced expression of ECS, that suggesting a possible indication of the therapeutic implication of cannabinoids in PD [426,427,428,429,430,431,432]. Compared to age-matched control, substantia nigra of PD patients displays loss in CB1 receptor availability. In contrast, the same study also demonstrated a significant increase in CB1R in three dopamine-rich areas, including nigrostriatal, mesolimbic, and mesocortical, respectively [432].

Pisani et al., demonstrated relatively higher expression levels of AEA in CSF of patients with PD, which was reached to normal following dopamine replacement therapy [431]. In parallel to these observations in an experimental model of PD treated with reserpine, 2-AG levels in globus pallidus were enhanced seven-fold and proposed to be associated with suppression in locomotor activity [433]. Furthermore, studies have also demonstrated changes including decrease in EC degradation and suppressed level of membrane transporter FAAH and AEA in the striatum of an experimental model of PD [428]. Taken in consideration, increased ECS in patients with PD was proposed to be associated with restoring striatal function to normal because increased CB1R reduces the release of glutamate, which is normally obtained by dopamine receptor 2 [426,434]. All these interconnected cascades of events resulted in blocking the direct pathway and the activation of the indirect pathway, which consequently leads to bradykinesia as well as distorted muscle movements characteristic of PD patients. At present, the best possible treatment to restore dopamine deficit in PD patients is L-DOPA; however, prolonged and sustained use of drugs is prone to lose its efficacy, and patients develop motor complications (dyskinesias) with long-term use. There are several direct and indirect evidence supporting the possibility of ECS association and use of cannabinoids in the treatment of PD, including the presence and colocalization of CBRs and dopamine receptors in substantia nigra pars compacta and striatum. Changes in CBRs expression and immunoreactivity in PD are scant and limited studies have shown the role of ECS in PD using Δ^9^-THC but concluded that Δ^9^-THC had no effect on PD [435]. On the contrary, in vivo experiments with laboratory animals have shown the potential to discover therapeutic uses of cannabinoids [435]. The endocannabinoid system, especially the CB_1_ receptors, interacts with the dopamine receptors; however, attempts to fully elucidate their biochemistry interaction are still underway [436]. The accumulation of proteins called Lewis bodies and the loss of dopaminergic neurons in the midbrain, specifically the substantia nigra are the prominent cause of PD. The activation of CB1R in the presence of either 2-AG or Δ^9^-THC, resulted in inhibition of Ca^2+^ and accounted for the reduction in GABA neurotransmitters [436]. Therefore, the activation of CB_1_ receptors may slow the decrease in dopamine due to PD [436]. Furthermore, results regarding cannabinoids obtained from the experimental model of PD are not conclusive but rather conflicting. In the reserpine quinpirole induced rat model of PD, reduced akinesia was further reduced upon treatment with WIN 55,212-2 as a specific agonist of CBRs, whereas this effect of WIN 55,212-2 was blocked in the presence of CB1 antagonist rimonabant (SR141716A) [437]. In the 6-OHDA induced model of PD, both Δ^9^-THC and CBD afford protection of dopaminergic neurons in substantia nigra, and in addition, Δ^9^-THC and CBD attenuate dopamine depletion and reduce expression of TH [438]. Previous studies also proposed inhibition of oxidative stress the effect that is associated with increasing antioxidants enzyme by induction of nuclear factor-erythroid 2 (Nfr-2) [439]. Growing evidence support the role of cannabis in suppression of multiple factors linked to perturbed neuronal functions and neurotoxicity in PD. Preclinical observations using cannabinoid including CE-178253, Oleoylethanolamine, nebilone and HU-210 have shown beneficial in suppression of reported bradykinesia and dyskinesia in PD patients following levodopa treatment [323].

Δ^9^-THCV has also been reported neuroprotective in the 6-OHDA model and LPS induced inflammation. This beneficial effect is possibly mediated through its antioxidant effects as well as through upregulation of CB2 receptors, which can therefore affect microglia activation. Also, in both models of PD, it was demonstrated that administration of Δ^9^-THCV in 6-OHDA induced lesioned and LPS induced neurotoxicity via involving glutamatergic transmission interfere in disease progression, reducing motor inhibition [268]. While in a rodent model of PD, cannabinoids have been implicated in therapeutic value whereas results are discouraging or confliction in 1-methyl-4phenyl-1,2,3,6-tetrahydropyridine (MPTP) lesioned primates model [427]. Qureshi et al., reported that, like an opioid, cannabinoids have also been supported for the use in PD-associated pain but with fewer adverse effects than opioids [440]. In addition, the role of cannabinoids in protecting mitochondrial damage and the role of glial cells in PD are of significant importance for future studies. However, the direct role of eCB in PD is not well studied and molecular mechanism linked to neurogenesis and PD are poorly defined despite studies proposing adult neurogenesis in the brain tissue of PD patients as well as in experimental model of disease. Moreover, growing evidence indirectly strengthens the significant contribution of neurogenesis in the pathophysiology of PD. In conclusion, considering previous studies, it seems results are very ambiguous regarding the protective role of cannabinoids in PD; however, cannabinoids can be a choice of drugs of preference to ameliorate the symptoms, including akinesia and tremor as well as motor abnormalities.

## 25. Multiple Sclerosis and Cannabinoid Receptors

Multiple Sclerosis (MS) is one of the most devastating common chronic CNS autoimmune diseases caused by demyelination with no treatment. At present, 2.3 million people are affected with MS worldwide, and the incidence of MS is often seen in the young adult stage, being that the most frequent age of MS is often diagnosed at the age between 20 and 40 years old [1]. Furthermore, epidemiological studies revealed that MS is prominent in women in comparison to men and geographically in northern locations. Although, the progression of the disease is still not well understood. Three well-connected events, including inflammation, demyelination, and axonal damage are often seen in MS. MS is a human autoimmune neurological disease caused by demyelination due to inflammation and the presence of immune cells. Although there are number of immune cells present, among them CD4 Th1 cells, in particular, have drawn great attention in the pathogenesis of MS. At present, IFN-β and Copaxone are the potential therapeutic drugs for MS with some limited success because they are not a curative option but have the ability to reduce relapses. The role of cannabinoids in the control of symptomatic MS and gradual progression of disease has been appreciated in recent studies. The neuroprotective role of cannabinoids is probably the best studied in the pathophysiology of demyelinated diseases, including MS. Although, there are many different ways to use cannabinoids, clinically in MS patients, depending on preparation, cannabinoids are often used as a standardized oral capsule and liquid sublingual spray. The administration of cannabinoids improves clinical and pathological abnormalities of MS and can avert disease symptoms and might exert a neuroprotective effect along with the interference in disease progression. Similarly, increasing AEA affords beneficial effects in MS. Cannabinoids govern immunomodulatory action, and clinical trials are in progress to determine the effect of Δ^9^-THC in MS [441]. Cannabinoid agonists have made progress in the Experimental Autoimmune Encephalomyelitis (EAE) model of MS and showed potent immunosuppressive properties of cannabinoid receptor agonist R (+) WIN55,212; however, such role of the cannabinoids is awaited in a Theiler murine encephalomyelitis virus (TMEV) model [441].

Although, the damage to myelin from cytokines released from autoreactive T cells and macrophages is the prominent cause of MS, disease progression from asymptomatic to the symptomatic stage also involves neuronal damage. A possible link between neuronal and immune systems is expected to be endocannabinoids, implicating the role of CB2R in regulating immune system and neuroprotection by involving CB1R [48]. The concept that cannabinoids can be used as a potential therapeutic target in MS emerged from the anti-inflammatory effect of cannabis and the ability of cannabis to suppress diverse immune cell functions. While the treatment of MS is still unknown, cannabis was the first drug used as an effective drug in inflammatory disease and can be an effective treatment option for MS. Pretreatment of an animal subjected to the EAE model with Δ^9^-THC associated with not only delayed the progression of the disease to the symptomatic stage but also reduced the severity of disease [442]. Furthermore, in support, mice devoid of CB1R resulted in enhanced neuronal damage in the EAE model. The use of CBD is approved in the UK and European countries for MS treatment [35].

### 25.1. Role of CB1R in Pathogenesis of Multiple Sclerosis

While CB2R is the major cannabinoid receptor subtype linked with MS pathology, previous studies revealed that CB2R mediated effects are not abolished completely in the presence of receptor-specific antagonists and, importantly, independent of CB1R [443,444]. Previous studies have shown proinflammatory cytokines and ROS in microglia, neurons, and astrocytes in CNS, probably in brain regions rich with CB1R expression, supporting the receptor’s role in MS. In mice model of MS, cannabinoids, through the anti-inflammatory effect of CB1R, are known to ameliorate disease progression [445]. The expression of CBR subtypes in oligodendrocytes for the first time emphasized the investigation in white matter and its association with MS.

### 25.2. CB2R and Implication in Multiple Sclerosis Pathogenesis

In an experimental mouse model of MS, microglial CB2 receptor activation provides a protective effect in inflammatory-induced CNS damage [264,446]. A wide range of clinical symptoms seen in patients with MS are associated with demyelination and perturbed neuronal function caused by the formation of inflammatory plaques containing immune cells. CBR subtypes 1 and 2 are present in immune cells; whether they contribute to the regulation of immune function in a comparable or competitive manner is not well understood. However, the expression of CBR, specifically CB2R in immune cells and its association with the pathogenesis of MS, establishes a link between CB2R and inflammation and the progression of MS. This section of this review describes different immune cells that might play a crucial role in the regulation of cannabinoid-mediated effects in MS.

### 25.3. Immune Cells, Cytokines, Inflammation, and Cannabinoids: Supporting Role of CB2R in Multiple Sclerosis

The molecular determinant for the role of CB2R in demyelinated diseases is not well understood; however, previous studies support the presence of CB2R in different types of cells of the immune system, including CD4 T cells, B lymphocytes, dendritic cells, astrocytes, and microglia/macrophages—suggesting that the immune response and anti-inflammatory role of these cells in response to cannabis are primarily regulated by CB2R. Previous studies have also described the distinct distribution of CB2R in human hematopoietic cells and mice [10,444,447].

### 25.4. CD4+ T Cells and CB2R and Immune Response and Multiple Sclerosis

The suppression of CD4 cells infiltration to the spinal cord in response to agonist-mediated CB2R activation in TMEV-IDD mice and inhibition of these cells from the peripheral site to CNS is the first indication of the role of CB2R in MS. Also, CB2R agonist and ligand-mediated inhibition of stromal cell-derived factor-induced chemotaxis and trans-endothelial cells migration support the role of CB2R [448,449]. Furthermore, CB2R-induced apoptosis in CD4 cells in the spinal cord is linked to inflammation. CD4 T cells of Th1 subtype have the ability to release several proinflammatory cytokines supporting the existing notion that CD4 cells are a possible cause of MS [450]. Taken into consideration, CB2R-mediated inhibition of cytokines from Th1 and Th2 cells and inhibition of CD4 migration to CNS account for the beneficial effect as well as suppressed survival of oligodendrocytes concomitantly involved in exacerbation of MS. Martin et al., proposed that expression of CB2R in immune as well as in neuronal cells might be involved in negative regulation of inflammatory response, one of the possible causes of demyelination in the pathogenesis of MS and experimental model [444].

### 25.5. CB2R, B Lymphocytes and Multiple Sclerosis

At present, the direct significance of CB2R in the pathogenesis of MS is elusive. However, with the role of CB2R in the maturation of B lymphocytes to plasma cells, which secretes the antibodies detected in MS as well as in the animal model of disease, and induction of apoptosis in lymphocytes has been reported [451]. Dendritic cells (DCs) are highly specialized and prominent antigen-presenting cells associated with the regulation of lymphocytes and are believed to be critical in the development of innate and adaptive immune systems [452]. Once recruited to CNS, the role of DCs is the regulation of autoimmune response and have a crucial role in the pathogenesis of MS and the animal model of disease [444,453]. Besides the activation of effector lymphocytes, DCs exert a crucial role in the regulation of inappropriate immune responses. DCs from MS patients displayed increased expression of IL-23 heterodimeric cytokines and are expected to initiate the response of Th1 [454,455]. Although, the precise role of CB2R in MS via modulation of DCs is not well understood. Previous studies have shown the role of CB2R in the migration of DCs to lymph nodes as well as apoptosis in DCs following activation of the receptor [456].

### 25.6. Microglia and Macrophages in Multiple Sclerosis and Role of Cannabinoid

The release of reactive oxygen and nitrogen species from microglia and macrophages in producing damage to myelin, neurons, and oligodendrocytes is well established. CB1R and CB2R are well expressed in microglial cells, and once activated, these cells can produce more endogenous cannabinoids compared to neuronal cells [48,457,458]. These cells are also able to release proinflammatory cytokines, specifically tumor necrosis factor (TNF-α), which is sufficient enough to exert damage to neuronal cells, probably involving excitotoxicity in addition to increased lymphocytes and increased expression of vascular cell adhesion protein 1 and intercellular Adhesion Molecule 1 [459]. Cannabinoids inhibit the release of cytokines via activation of CB2R and inhibition of inducible NOS as well as nitrites production in response to ECs blocker UCM707, CBRs agonist WIN 55212, and anandamide in a CBR-dependent manner supporting the role of cannabinoid in MS. Furthermore, glutamate induced neuronal loss is also seen due to increased Ca^2+^ through the activation of NMDA receptor upon suppression of IL-β [460].

### 25.7. CBR Subtypes and Astrocytes: Association with Pathogenesis of Multiple Sclerosis

CBR subtypes are expressed in astrocytes and elicit both harmful and protective effects in CNS [136]. Astrocytes have the ability to produce NO due to the presence of inducible nitric oxide synthase on them, and cannabinoids inhibit nitrite release from astrocytes through the activation of CB2R [136]. Furthermore, they also inhibit inflammation induced by the release of cytokines, including TNF-α, IL-1β, and IL-6 from astrocytes, which are inhibited by cannabinoids through the activation of CB1R and CB2R [461]. In a viral model of MS, cannabis affords protection of axonal damage due to excitotoxicity with expression of IL-6 and block the progression of MS with possible activation of CB2R [462].

## 26. Amyotrophic Lateral Sclerosis and Cannabinoid Receptors

The loss of antioxidant enzymes prompts excessive production of ROS and weakens body’s defense against oxygen free radicals. However, the mutation in one of the antioxidant enzymes, namely superoxide dismutase-1 (SOD-1) caused Amyotrophic Lateral Sclerosis (ALS), a rapidly progressive neurological disease with the loss of motor neurons that consequently resulted in muscle denervation, atrophy, and paralysis [463,464]. In addition to mutations in SOD-1, the mutation in TAR-DNA binding protein-43 (TDP-43) or fused in sarcoma (FUS) protein has also been reported [464,465]. Furthermore, recent studies have also reported hexanucleotide (CCG GGG) expansion in C9orf72, a possible cause of ALS [463]. Familial cases of ALS constitute only a small population, whereas large numbers of ALS patients are sporadic. Like many other neurological diseases, the degeneration of motor neurons in ALS also involves excitotoxicity, proinflammation, oxidative stress, protein aggregation, and other cellular signaling to induce neurotoxicity [463,465,466,467]. Individuals suffering from ALS often exhibit motor and cognitive deficits.

At present, besides riluzole, an approved drug with anti-excitotoxicity properties, no other effective treatment is available for ALS. The expression levels of ECS, including receptor expression, and levels of eCB metabolizing and degrading enzymes in ALS patients and in mutant animals, are the first evidence supporting the role of cannabinoids in ALS. Previous studies using a transgenic mouse model with overexpression of mutated SOD-1 as well as mice lacking FAAH and CB1R, worsened the disease. The brain regions, including the spinal cord, brain stem, and cortex, are affected the most in ALS. In this context, studies using SOD-1 mutants have demonstrated increased expression of AEA and 2-AG in the spinal cord [468,469]. Along with these observations, increased expression of anandamide synthesizing enzyme without any changes in the enzyme which synthesizes 2-AG as well as degrading enzymes FAAH and MGAL [470]. However, the role of CB1R in ALS is not conclusive, contrary to increased expression of CB2R in microglia in ALS patients [471] as well as in SOD-1 mutant and TDP-43 *tg* mice [470,472,473]. In addition to the results from a mutant and transgenic model of ALS, limited clinical observations support the neuroprotective role of cannabinoids in ALS. Although evidence is still lacking to support of cannabinoids as a disease-modifying therapeutic intervention, Δ^9^-THC exhibits improved ALS symptoms using SOD-1 mutant mice, described that Sativex affords protection of motor neurons but fails to defend neuron–muscle joint without any noticeable improvement in animal survival time [470]. The cannabinoids mediated neuroprotective effects in ALS are proposed by three different mechanisms first, CB2R-mediated reduction in microglial activation and neuroinflammation with the possible association of PPAR-γ second, CB1R-mediated reduction in excitotoxic damage; and third, receptor-independent antioxidant effects of cannabinoids that might be related to PPAR-γ/Nrf-2 signaling [474]. Although, significant progress has been made to understand the medicinal use of cannabinoids in clinical trials; however, benefits are marginal or inconclusive due to the limited sample size. In conclusion, from the studies discussed above, cannabinoids afford a beneficial role in ALS, including protection against excitotoxicity and oxidative stress as well as the suppression of proinflammatory signaling, including the release of cytokines via microglial inactivation.

## 27. Therapeutic Implications of Cannabis in the Treatment of Neuropsychiatric Disorders

The term “cannabis psychosis” emerged from the occurrence of psychosis with the use of cannabis in some users. Early studies revealed that modes of application are associated with cannabis-induced psychosis. The relation between cannabis and schizophrenia is undisputed. Although significant progress has been made, the understanding of molecular determinants of this complex and heterogeneous pathogenesis of schizophrenia with the use of cannabis is not well understood and leads to poor diagnosis and prognosis. It is believed that among schizophrenic patients, cannabis is the most common choice of substance abuse in comparison to other drugs. Furthermore, the risk factors with the use of cannabis have been reported in a dose- and time-dependent manner [302,475,476,477]. Psychotic patients are prone to relapse with the use of cannabis, and individuals with ongoing treatment with antipsychotics are also highly vulnerable to the acute effect of cannabis [302]. Often, studies noticed that CBR agonists, specifically synthetic cannabinoids, prompt higher levels of aggression and agitation, which are often seen with severe psychotic episodes [478]. The modulation of a high-risk pattern of psychotic disorders and the incidence of relapse with the use of cannabis can be well associated with inflammation [479]. The presence of CBRs in immune cells is further supported in psychotic patients with the use of cannabis as an indication of regulation of the immune system [30]. The exogenous use and receptor-induced release and activation of endocannabinoids, specifically AEA, have potentiated and reduced schizophrenia symptoms, including negative and positive symptoms. These observations support the intimate association between schizophrenia/psychosis and cannabis; however, the molecular mechanisms involved are still poorly understood. Several experimental approaches either addressing the molecular details, or pharmacology, or neurochemistry, support that heavy use of cannabis is certainly associated with schizophrenia symptoms and also may exacerbate these symptoms in individuals with psychosis. In control naïve individuals, short-term use of cannabis resulted in psychotic symptoms and cognitive deficits [113,480,481]. Furthermore, studies also revealed inflammation as a possible link between cannabis and psychosis [302].

Schizophrenia is a neuropsychological disorder that happens to be an idiopathic disease [482]. Schizophrenia’s symptoms are mostly neurological; for example, people who suffer from Schizophrenia display instability, delusion, and often hallucination [482]. The molecular details of biochemical, neurochemical, and molecular changes that occurred in Schizophrenia are not well understood; however, cumulative studies speculated three plausible causes for Schizophrenia—genetic, environmental, or even social [482]. Recently, an excellent review by Borgan et al., on CB1R and schizophrenia summarized from collective epidemiological, preclinical, and experimental studies detailed that endocannabinoids might be associated with the pathogenesis of schizophrenia with a significant therapeutic contribution of CB1R [113]. This review further dissected some key observations from preclinical and animal studies in support of CB1R’s mediated crucial role in this devastating psychological disorder. Furthermore, at the molecular levels, a triplet repeat polymorphism in the CB1 gene has been associated with hebephrenia, a cognitively impaired phenotype of schizophrenia in human [356,483].

Cannabinoids play an essential role in creating the architecture and wiring of the brain. In this section of the review role of cannabinoids is discussed with different hypotheses linked to the progression of negative and positive symptoms. The complexity of schizophrenia is first negative and positive symptoms; second is the changes in multiple neurotransmissions, including members of the GPCR family and ionotropic glutamate receptors. In the pathogenesis of schizophrenia, DA, GABA, adenosine, serotonin, and metabotropic glutamate receptors have been proposed as crucial determinants [484,485]. In addition to the significant contribution of changes in GPCRs and ionotropic glutamatergic neurotransmission, and the role of inflammatory insult has also been associated with schizophrenia. Moreover, the immunomodulatory properties of cannabis further support the role in pathogenesis and inflammation in psychosis. Interestingly, psychosis associated with most inflammatory abnormalities is regulated by cannabis, specifically through the CB2R subtype.

## 28. Cannabinoid Hypothesis of Schizophrenia: Schizophrenia and Use of Cannabis

Several previous studies have been witnessed to support the causative relationship between cannabis and psychosis for a long time. Increased risk and early onset of psychotic disorders have also been reported with the use of cannabis in addition to psychotic effects in healthy people with the use of Δ^9^-THC and synthetic cannabinoids [486]. The increased expression level of endocannabinoid, i.e., AEA, is frequently seen in CSF of patients suffering from schizophrenia and in individuals at risk for psychosis as well as in patients with first episodes of psychosis [487]. Furthermore, the person who has not received any medicine and never used cannabis also exhibits increased levels of AEA in CSF with the occurrence of the first episode of psychosis [488]. Palmitoylethanolamide, an endocannabinoid that binds to CB2R, is also increased in schizophrenic individuals. This is worth mentioning here that increased endocannabinoid is not suppressed in a patient with medication, which further emphasized that an enhanced level in CNS is the possible cause associated with the pathogenesis of schizophrenia. The role played by cannabis in psychosis is controversial and has also been associated primarily with the expression level of endocannabinoid AEA despite the fact that the psychoactive gradient of *Cannabis sativa* L. Δ^9^-THC prompts similar symptoms as seen in schizophrenia [113]. Growing evidence supports the idea that the endocannabinoid system may substantially contribute to the causes of some symptoms of Schizophrenia. Although the psychotic disorder is seen following consumption of a large number of cannabinoids, specifically Δ^9^-THC, often precipitate a psychotic state, also known as cannabinoids psychosis, that governs a different mechanism than the exact neurological disease but attests to the role of cannabis in schizophrenia. Second, functional changes in CBRs are also associated with the pathogenesis of schizophrenia. Further studies of the interaction of cannabinoids with other pathways may provide a new therapeutic approach to Schizophrenia. It is also believed that cannabis use may lead to the precipitation of psychosis in individuals who are susceptible to psychosis. Previous studies also support that early exposure to cannabis has a high chance of developing schizophrenia, and individuals with preexisting schizophrenia with the use of cannabis often enhanced the severity of the disease and the chances for relapse [489,490,491,492,493]. Leweke et al., proposed that inhibition of anandamide deactivation is a possible contributing factor that is associated with cannabidiol mediated antipsychotic effect and suggested CBD as a new therapeutic intervention in schizophrenia treatment [494]. In contrary, studies have also revealed no effect of CBD in schizophrenia [495,496]. At the molecular level, studies have shown susceptibility to schizophrenia with certain alleles or genotypes in the CNR1 gene [483].

### 28.1. The Dopamine Hypothesis of Schizophrenia and the Role of Cannabinoids

The most prevalent notion supporting schizophrenia is the imbalance in dopaminergic neurotransmission in patients with schizophrenia, and dopamine sensitization is often seen in acutely psychotic patients [235,497,498,499]. The interaction between dopaminergic and cannabinoid systems and the role of enhanced DA in psychosis is well established. Furthermore, cannabinoids can be a possible cause of psychosis and also worsen the condition. Activation of DR subtypes seems to correlate with the increased AEA levels [500]. Further, in support, studies have shown hyperactivity of dopaminergic transmission, which promotes the positive system, and DRs antagonists, which inhibit DRs. Specifically, D2R exhibits positive symptoms but not negative symptoms and is also unable to improve cognitive deficit in patients with schizophrenia [501,502]. Furthermore, the limitations of this hypothesis include some side effects with the use of D2R antagonists and a second weak connection with symptoms and clinical diversity. Therefore, it is speculated that there must be numerous other pathways involved with schizophrenia [500]. Furthermore, other neurotransmitters, including glutamate and serotonin, also regulate the dopaminergic system and are expected to participate in schizophrenia. The dopamine hypothesis in schizophrenia in association with cannabinoids is further strengthened by dopamine and AEA functional interaction. Previous studies revealed selective and preferential stimulation of D2R but not D1R type in response to the release of AEA in the striatum with expected modulation of DA-induced psychomotor activation as suggested. In contrary to AEA, 2AG, and N-palmitoylethanolamine (PEA) [49], cannabis is also associated with increased release of DA in the striatum and neuronal firing [503,504]. Although human data are limited; however, studies have shown increased synaptic Dopaminergic activity in response to cannabis [235,505]. Multiple interconnected events include the overactivation of the dopaminergic system, the relevance of dopamine transporter knockout mice with schizophrenia, reduced AEA in the striatum, and most antipsychotic drugs target dopaminergic systems support the dopaminergic hypothesis in concert with cannabinoids [506].

### 28.2. NMDA Receptor Hypothesis of Schizophrenia with the Implication of Cannabinoids

Swinging mood behavior is often linked to an imbalance in NMDA function and is considered a critical determinant of individual behavior. The previous finding that NMDA activity exerts a stimulatory effect on endocannabinoid release established the first association of the NMDA receptor hypothesis in schizophrenia in concert with cannabinoids via CB1R. Suggesting that CB1R ability to limit NMDA function may suppress NMDA-mediated excitotoxicity [403,490,507]. Furthermore, NMDA receptor antagonists have an ability to mimic prominent symptoms of diseases, including positive, negative and cognitive changes supporting the role of glutamatergic pathway in schizophrenia.

### 28.3. Neurodevelopmental Hypothesis of Schizophrenia

An individual suffering from schizophrenia often exhibits gradually reduced brain size. Whether cannabis use is associated with suppression of brain size is not confirmed, and reduced and normal brain size have been reported [508]. Endocannabinoids mediated a crucial role in neurogenesis, supporting the neurodevelopmental hypothesis of cannabinoids. Previous studies have shown the potential role of neuregulin and ErbB4 receptor genes in neurogenesis and their role in developing schizophrenia [509]. Furthermore, the susceptibility of mice to develop psychotic effects with Δ^9^-THC and enhanced induction of cFos expression with hypomorphic neuregulin support the concept of this hypothesis [510,511].

### 28.4. Independent Risk of Schizophrenia

It is well understood that in Schizophrenic patients, there are considerable changes in dopamine and glutamine levels compared to normal individuals. Also, morphological changes, including increased ventricle sizes, and decreased masses, are also seen in schizophrenia. In addition to different hypotheses as discussed above, some of the changes at the molecular level have also been reported in Schizophrenia which may account as an independent risk factor of disease. Heterogenous population with schizophrenia revealed genetic component as a critical factor associated with disease and many other genes, including catechol-O-methyl transferase (*COMT*), dysbindin (*DTNBP1*), a regulator of G-protein signaling 4 (*RGS4*), disrupted-in-schizophrenia 1 (*DISC1*), and metabotropic glutamate receptor 3 (*GRM3*, also known as *MGLUR3*) and neuregulin1 and its receptor ERbB4 are linked to schizophrenia [509,512,513]. The significant progress have been made in the last few years regarding the increased risk factors which attests to the crucial role of polymorphism in neuregulin and ERbB4 and its consequences on neurotransmission and synaptic plasticity [509]. Furthermore, changes in glutamatergic and GABAergic neurotransmission and synaptic plasticity are often observed in schizophrenia, and neuregulin in the adult brain is involved in the regulation of excitatory and inhibitory neurotransmission [509,514,515,516]. Although no direct evidence for the role of cannabinoids in the independent risk of schizophrenia has been reported yet, with the changes in glutamatergic and GABAergic neurotransmission, the role of cannabinoids cannot be avoided.

## 29. Role of Cannabinoids in Depression, Stress, and Anxiety

A psychiatric condition in individuals is characterized by continuous loss of interest, whereas anxiety is associated with feeling of fears, and both depression and anxiety often co-occur. Whether or not these two mood disorders are two different entities or one is controversial. Depression and anxiety have been associated with individuals of all ages and are increasing risk factors for other disorders. Growing evidence supports that ECS is involved in regulation of these mood disorders or behavioral changes. This section describes the use of cannabis as a therapeutic alternative in treatment of these mood disorders. “Relaxation” is believed to be one of the prominent reasons behind the use of cannabis. Fear, anxiety, and stress associated with abnormal behavior is an inappropriate response to external and internal stimuli, which results in an imbalance between endocrine and neural responses. In this context, ECS exerts a determinant role in the regulation of behavioral changes through the modulation of synaptic transmission [517]. However, this pathway is more complex but well connected between different brain regions and peripheral systems through the hypothalamic–pituitary–adrenal axis (HPA) and the sympathetic system [517]. Whether the HPA is a central control of eCB association with stress and anxiety is not conclusive yet; however, a dysfunctional HPA involved in psychiatric disorders is well established and the stress-associated release of CRH regulating eCB and receptors supports the notion. The inhibition of HPA activity is observed in response to eCB-mediated signaling that resulted in suppression of glucocorticoid levels, consequently linking HPA activity to acute stress. However, long term changes have also been reported with low glucocorticoids as well as upon activation of HPA supporting a feedback-dependent regulating role of eCB [517]. Aging is a critical factor in HPA response to stress and it is proposed that adolescence exhibit greater sensitivity in HPA than adults. Furthermore, CB1r in association with AEA in basolateral amygdaloid is associated with gate behavior and neuroendocrine response to stress and fear promoting stimuli [517]. It should also be noted that stress-related behavior can lead to anxiety is associated with HPA sensitivity in response of reduced level of AEA. Moreover, a mechanistic association between eCB and stress as well as anxiety is also proposed through corticosteroid releasing hormone. In the amygdala and prefrontal cortex, increased levels of CRH in response to corticosteroids or stress is associated with modulation of eCB. Upregulated corticosteroid releasing hormone leads to the induction of FAAH, consequently leading to suppressed levels of AEA and 2-AG in the amygdaloid and also AEA expression in the prefrontal cortex. Taken in consideration, a crucial link is well established between stress, anxiety, eCB, and HPA.

The role of cannabinoids in stress is of several folds directly or indirectly either linked to the central or peripheral CBR subtypes. In response to acute stress, endocannabinoids provide relief from stress-related behavioral changes and stress endocrine disturbances. Henquet et al., proposed a cross sensitization between stress and cannabis because previously exposed individual to stress respond to additional stress to the greater extent in overtime [518]. Furthermore, the role of stress as a risk factor in psychosis linked to the use of cannabis cannot be avoided. CBDA prevents a stress-associated anxiolytic effect as well as suppresses stress-induced anxiety and pain. CBD, a non-psychotic cannabinoid averts stress associated neurogenesis as well as neurogenic abnormalities seen in the experimental mouse mode of AD [392,519]

**Figure 9:**
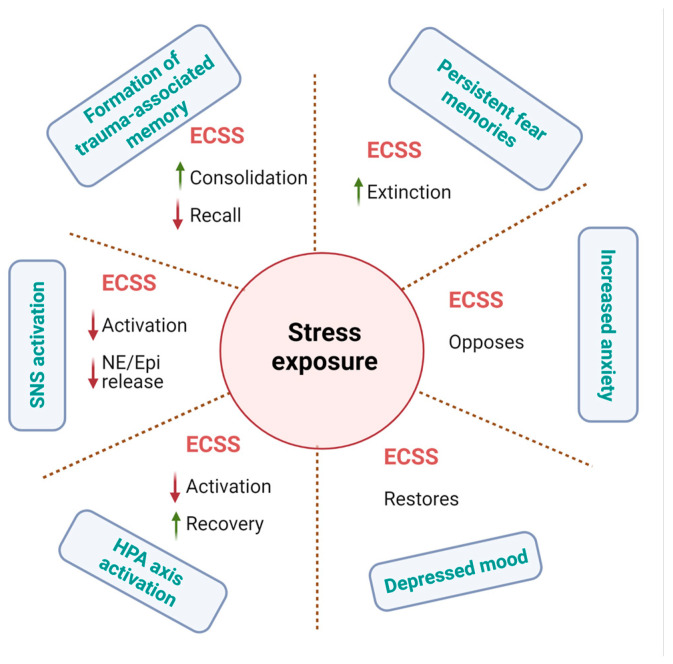
Schematic illustration showing stress associated abnormalities and role of cannabis. This figure was created with BioRender.com.

Stress, a mental tension that arises in a difficult condition, is a growing concern worldwide and poses a significant financial burden in addition to enormous social and healthcare responsibilities. Although stress is individual, multiple molecular and environmental factors are linked and represent a complex process of origin without any effective treatment available yet (Figure 9). The expression of ECBs in brain regions associated with stress, activation of the HPA, and glucocorticoid release involved in regulation of hormonal/endocrine response to stress attest the crucial role of ECBs in stress response and several other brain functions. Furthermore, the activation of HPA is well associated events in the process of stress [520]. Hill et al., reported disinhibition of neuronal loss in the basolateral nucleus due to increased activity of FAAH and suppressed levels of AEA and changes in HPA [521]. The role of cannabinoids as a therapeutic alternative for stress treatment is well established; however, the molecular mechanism of regulation of physiological and behavioral responses of stress is not well defined. Meanwhile, the consequences of acute and chronic use of cannabis and occurrence of stress are not well understood. The use of cannabis begins at early stage of life often leads to impaired cognitive function and stress and resulted in psychological disturbances. Studies have also shown that cannabinoids worsen the effect of early life stress [522]. Cannabinoids are known as an effective therapeutic alternative for the treatment of stress and associated other behavioral changes. However, the role of cannabis used at early stage in life to cope with stress is different than cannabis used at adult stage for the similar pathological conditions. Alteba et al., proposed the pervasive and negative effect of cannabinoid use during adolescence, but it may exert a protective effect against an early-stage stress-induced devastating effect if used at late adolescence with some significant age-dependent implication of cannabinoid as therapeutic intervention [522]. Stress is one of the crucial reasons for instigating the use of cannabis and in parallel to the increase in cases of stress the numbers of cannabis user are gradually increasing [523]. In contrast to these observations, an increasing number of stress-associated behavioral changes can be seen. Taken into consideration, existing results implicate not only the endocrine system through HPA but also involve excitatory glutamatergic and inhibitory GABAergic neurotransmission in complex process of stress in response to cannabinoids.

The incidence of association between cannabis use and depression has been reported regardless how often cannabis is used. Van Laar et al., from their three-year survey, reported that the occurrence of major depressive disorder in people with cannabis use is higher than non-cannabis users, in contrast to the observations that cannabis users are not at risk for developing the major depressive disorder [524,525,526]. Furthermore, accumulated information from the WHO 2016 and National Academy of Science, Engineering, and Medicine 2017, USA, summarized no association between cannabis use and depression. The use of cannabis as a clinical approach to treat depression is controversial and supported by conflicting observations from previous studies [527,528]. Depression is the most common brain illness and affects a large population worldwide. Like many other neurological diseases, depression is also associated with changes in multiple neurotransmission as well as different pathways in the brain. Furthermore, in view of widespread distribution and multiple functions in CNS, it is imperative to study the interaction of cannabinoids with other neurotransmitters to better understand the use of medicinal cannabis and associated molecular mechanisms [529]. In an individual with depression, the impaired function of serotonergic transmission and perturbed synaptic function is generally observed, and such changes are thought to be linked with an increase in NMDAR function [490,530,531,532]. At the molecular level, rodents that are CN1R gene-deficient are prone to depression symptoms [533], which further correlated with suppressed endocannabinoid signaling in the brain in the rodent model of depression [353,534]. In contrast, depression can be a possible inducer or factor for cannabis use because individuals who use cannabis frequently seem less depressed, with more positive effects than non-cannabis users [535]. There is some evidence that establishes a connection between cannabis and depression. It is often observed that cannabis use for pleasure abolishes the feeling of negative, depressive behavior, and in these individuals, signs of depression do not occur [536]. Previous case studies also argued that the use of cannabis in depressed individuals enhanced the antidepressant effect [537,538]. Moreover, studies have also established some relation between low levels of endocannabinoids and depression [521,539].

In addition to depression, anxiety and stress might be a reason to move toward cannabis use. The role of Phyto-CBs is well recognized in regulating perturbed social behavior. The role of cannabis in anxiety is controversial, and as a result of a lack of sufficient studies, it is not well defined; furthermore, most studies performed so far are dealing with this in MS patients or any other psychological abnormalities but lacking a sample with an anxiety diagnosis. However, emerging evidence from pharmacological and molecular studies supports the role of endocannabinoids in anxiety. In addition to the psychoactive effects with the use of cannabinoids, anxiety-like properties are also described. Studies have shown that cannabinoids interact with endocannabinoids and are involved in the regulation of behavioral responses. Although the increased risk of psychosis with the consumption of cannabinoids is undisputed, to what intensity cannabis is involved in anxiety is elusive. Nevertheless, answers to some clinical questions are still awaited. Botsford et al., in their recent review, made available some excellent observations regarding the role of cannabinoids and anxiety or, in a broader sense, on mood disorders [540]. The authors summarized that only 3 cases out of 12 develop anxiety symptoms. Child et al. described the dose dependency of oral Δ^9^-THC use and indicated that low doses of Δ^9^-THC are anxiolytic, whereas, at higher doses, Δ^9^-THC governs anxiogenic properties [541]. In conclusion, it seems that future studies are required to explore the therapeutic importance of cannabinoids in anxiety. Endocannabinoids are well expressed in the brain region, which plays a crucial role in anxiety, stress, and fear. Furthermore, studies from CB1R knock-out mice showed increased anxiety behavior with aversive conditions [517]. This is important to note that the presence of CB1R on cortical glutamatergic and GABAergic neurons plays an opposing role in the regulation of anxiety [517]. Furthermore, the dose-dependent effect of cannabinoids depends on the presence of CB1R on glutamatergic or GABAergic neurons in the cortex. Rubino et al., showed that when Δ^9^-THC is administered to the hippocampus or prefrontal cortex at low doses exerts an anxiolytic effect contrary to the anxiogenic effect at high doses [542]. These observations emphasized the role of excitatory and inhibitory input and its association with the opposing effect of cannabinoids on anxiety. Furthermore, the role of endocannabinoid synthesizing and degrading enzymes has also been associated with anxiety [517]. Growing evidence also supports the role of CBD as a stress suppressor as well as in fear expression and panic disorder. The use of CBD in humans with a social anxiety disorder is also known to suppress anxiety through specific brain regions, including limbic and paralimbic [543]. It is worth mentioning here that AEA is also known to activate transient receptor potential cation channel subfamily V member 1 (TRPV1), and mice with TRPV1 ablation exhibited decreased anxiety-like behavior. In conclusion, cannabinoids via activation of CB1R play an important role in the regulation of anxiety. Most importantly, the association of CB1R with the glutamatergic and GABAergic system further differentiates the biphasic effect of cannabinoids on anxiety-like behavior, as discussed above.

## 30. Cannabinoids and Acute Brain Damage: Stroke and Brain Trauma

Stroke is one of the most common causes of death and disability across the globe. Growing evidence supports that the causes, duration, localization, and severity of the injury and the presence of comorbidity factors are the critical determinants to impact the overall outcome and likelihood of survival. Stroke is further divided into two different types, including Ischemic and hemorrhagic. An ischemic stroke happens due to the blockage of blood flow through the artery that supplies oxygen-rich blood to the brain. A hemorrhagic stroke occurs when a blood vessel in the brain ruptures or leaks, causing bleeding into the brain tissue. More than 80% of strokes are ischemic, and hence, the everlasting search for new therapeutic targets amenable to pharmacological manipulation for ischemic stroke patients does not end. Following an occlusion, ischemic stroke is triggered by excessive release of glutamate, which further leads to cell death by causing an imbalance in the functioning of Ca^2+^ dependent enzymes, mitochondrial damage, and apoptosis. Traumatic brain injury (TBI), a leading cause of disability in young worldwide, is another kind of brain injury that is very similar to stroke (shares a similar pathophysiology) and happens when a sudden trauma affects the brain regions. Although therapeutic choices to treat stroke are limited, and the only available treatment is the recanalization of the occluded vessel along with thrombolytic therapy. However, very few patients can receive (<5%) due to its narrow time window. Multiple studies argue in favor of CB1R and CB2R contribution in brain injuries associated with trauma [544,545,546]. Furthermore, suppressed brain edema and inflammation has also been reported by endocannabinoids released post traumatic brain injuries [544,545,546]. Lopez-Rodriguez et al., using animal model of trauma described the opposing effect of CB1 and CB2R antagonist on drugs mediated neuroprotective effects, which supporting the neuroprotective effect of cannabis in traumatic brain injuries [547].

### Role of Cannabinoids in Stroke

The ECS has emerged as a new therapeutic target in a variety of neurological disorders due to its robust anti-inflammatory role in brain tissue injury. Endocannabinoid levels are known to increase in selected brain regions following neuronal damage, which may reflect a self-neuroprotective response [548]. In addition to thrombolytic therapy, a recent meta-analysis by England et al. stated that all subclasses of cannabinoids, including, cannabis-derived Phyto-CBs, synthetic, specific CB1R, and CB2R agonists, significantly reduced infarct volume in transient and permanent ischemia [549]. CBD is known to mediate neuroprotective effects through multiple mechanisms, including a combination of potent antioxidant, immunosuppression, and anti-inflammatory actions. Brain edema and blood–brain barrier permeability associated with the ischemic condition are also proven to be reduced by CBD [550]. Excitotoxicity following a brain injury can further potentiate cell death, and previous attempts have been made to attenuate the effects of excitotoxicity by targeting NMDA receptor antagonists. However, NMDA receptor antagonists showed promising results in pre-clinical models of TBI [551] but failed to produce long-term beneficial outcomes in clinical trials. Interestingly, in a rat model of fluid percussion injury, the CB1R antagonist, rimonabant reduced mGluR_5_ receptor expression preventing long-term hyperexcitability by up-regulation of dynorphin-κ opioid receptor (KOR) system 6 weeks following TBI [552]. This suggests that acute administration of cannabinoids post-injury may lead to long-term changes in glutamatergic function. Studies have also suggested that excitotoxicity leads to the elevations of anandamide with little or no changes in 2-AG [553,554]. Furthermore, in concussive head trauma in rats, increased anandamide levels occur in the ipsilateral cortex, with no change in 2-AG levels [554]. Tchantchou et al. also found a 1.5-fold increase in anandamide levels at 3 days post-TBI in ipsilateral mouse brain [553]. On the contrary, closed head injury in mice significantly elevated the level of endogenous 2-AG [555]. Further studies demonstrated that these elevations are endogenous responses addressed to limit brain damage, as the inhibition of anandamide and 2-AG hydrolysis reduced brain damage and improved functional deficits in parallel to a reduction in proinflammatory responses in the mouse brain after TBI [553,556]. The shearing and tearing forces of TBI, followed by secondary injury, produce changes in cell architecture, extracellular matrices, and the balance of fluid homeostasis that impair neuronal function throughout the brain [557]. While a traumatic insult can result in the rapid onset of cerebral edema, exogenously administered 2-AG protects against TBI-induced edema in mice [546,558]. No edema protection was observed following 2-AG administration in CB1R knockout mice [546,558], suggesting the role of CB1R activation in neuroprotection and neurorepair.

Dexanabinol, also known as HU211, is the only reported cannabinoid to be specifically evaluated for the treatment of TBI. While HU211 showed promising outcomes in pre-clinical studies [559], it failed to produce long-term patient outcomes in clinical trials despite some acute benefits [560,561]. Although HU211 has been described as a cannabinoid by virtue of it being an enantiomer of the potent synthetic cannabinoid agonist HU210, it does not bind or activate cannabinoid receptors. Instead, HU211 acts as a non-competitive NMDA receptor antagonist.

In parallel to beneficial effects with the use of cannabinoids, the deleterious effects of cannabinoids in stroke also exist and need to be discussed here. Several studies in animal models, as well as detailed meta-analysis observations, support the protective mechanism of the use of cannabinoids in stroke [549]. However, this concept has been challenged with studies from Shearer et al., describing the damaging effect of 2-AG after artery occlusion in a rat model of stroke [562]. Shearer et al., observations and associated mechanisms that might involve harmful effects have been discussed earlier [562] (see for details Pires 2018) [563]. Shearer et al., further emphasized that increased platelet aggregation is associated with harmful effects [562]. Furthermore, studies have also reported that cannabis use is associated with the risk of stroke independent from other factors such as smoking, alcohol and obesity [564,565]. Important observations are the incidence of ischemic stroke in the young population with the use of cannabis. Further studies from Rumalla et al. reported an increased risk of acute ischemic stroke in individual aged group of 25–34 years who use of cannabis [566]. On the contrary, no association between cannabis use and stroke was established by other [567]. In addition to platelet aggregation, mitochondrial damage associated respiratory chain function, and oxidative stress are also associated with ischemic stroke with the use of cannabis.

## 31. Role of Cannabinoid in Brain Tumor Glioma

The presence of CBR subtypes and the potent effect of cannabinoids in inhibiting cell proliferation and angiogenesis in various tumors, including brain tumors, support the role of cannabinoids in tumors of CNS origin. The role of cannabinoids in different types of cancer, specifically in chemotherapy-associated side effects, including nausea, vomiting, pain, and appetite, is well established. However, the role of cannabinoids directly in tumor pathogenesis of different origins, specifically in tumors of the CNS is not well understood. The antiproliferative role of cannabinoids via induction of apoptosis has been implicated in the suppression of tumor growth. In addition, cannabinoids have also been reported in cell death via induction of autophagy [568]. Also, changes in ECS have been reported in a tumor-specific manner. This section of the review focuses on the role of cannabinoids in the development of glioma and glioblastoma, which are the most frequent type of brain tumor, characterized by aggressive glial cell differentiation.

Glioma is declared one of the most aggressive and frequently occurring classes of brain tumors by WHO, with a short life post-diagnosis due to a high rate of proliferation and invasiveness [569]. Gliomas exhibit significant variations in molecular and cellular physiology, which are linked to high malignancy and recurrence as well as invasiveness. The concept that glioma originated from differentiated glial cells has recently been challenged with the observations describing the isolation of glioma-derived stem cells responsible for the malignancy of glioma. Studies reported apoptosis and inhibition of angiogenesis are two possible molecular determinants or mechanism that interferes with the progression of glioma [570,571,572,573,574,575,576,577,578,579,580]. In this context, the activation of CBRs induces apoptosis and inhibits angiogenesis. CBRs are expressed in most tumor cells and inhibit the proliferation or differentiation of normal progenitor cells [581,582]. In contrast to having a protective or no effect in cells of neuronal origin, such astrocytes, oligodendrocytes, and neurons, cannabinoids have been reported to mediate inhibition of cell proliferation and angiogenesis [91,571,573,578,583,584,585]

CB1 and CB2 receptors are both present in rat and human glioma [573,578,586,587]. CB1 and CB2 both displayed positive responses to Δ^9^-THC treatment as well as synthetic agonists, including WIN-55,212-2 and the CB2-selective agonist JWH-133. Previous studies have also demonstrated apoptosis and inhibition of angiogenesis following treatment with cannabinoids [569,573,587]. The first indication in support for the anti-tumor effect of cannabinoids in glioma emerged from studies describing epidermal growth factor receptors (EGFR) ligand amphiregulin increases the resistance to Δ^9^-THC antitumor effect in glioma xenograft [257,588]. EGFR, one of the receptor tyrosine kinase, plays a crucial role in tumor progression, resistance, and treatment failure. Δ^9^-THC is known to induce apoptosis via activation of CB1R and CB2R. It is interesting to note that apoptosis induced by cannabinoids is only at higher concentrations when used for a sustained time, whereas, at lower doses, cannabinoids prompt the proliferation in association with EGFR [587,589,590]. In glioma, cannabinoids also inhibit angiogenesis, and the anti-angiogenesis effect of cannabinoids in a glioma xenograft and inhibition of VEGF production in glioma in vitro and in vivo is well known [257,571,573,583]. Blazquez et al., reported the inhibition of adhesion, migration, and invasiveness in glioma and other tumor cells in vitro [591]. Cannabinoids are also known to block tumor growth and angiogenesis associated with the promotion of apoptosis, suppression of proangiogenic factor, and MMP production [573,578,583]. Furthermore, studies have also suggested inhibition of AKT–mTOR1 axis or stimulation of autophagy and apoptosis at higher concentrations of Δ^9^-THC that are used to glioma cell death [257,568,592]. Moreover, pharmacological blockade of CB1R and CB2R prevents cannabinoid-induced cell death [593], and studies have also determined the gene associated with Δ^9^-THC resistance but not the expression levels of CB1R and CB2R [593]. Blazquez et al., further emphasized the significance and contribution of angiogenesis in glioma progression and characterization [571]. The hypoxic nature of glioma is associated with dense vascularization and the proliferation of malignant cells. EGF-associated pathways and angiopoietin 2 have been used as a point of differentiation between grade IV astrocytoma, i.e., glioblastoma multiform from lower-grade astrocytoma [571,594,595,596]. Cannabinoids block the production of VEGF and its mediated signaling. In this way, inhibition of VEGF expression and activation of VEGF-R2 in response to cannabinoids in- vitro was proposed as a possible mechanism in support of the in vivo role of cannabinoids in tumor suppression. Furthermore, cannabinoid is known to inhibit EGF and NGF, which activate VEGF expression [571,597,598]. In addition, cannabinoid-mediated modulation of sphingolipids metabolizing pathways, which increases ceramide, may further involve the induction of apoptosis [599]. In this context, it has been proposed that CB2R activation supports a pro-apoptotic role by inducing the intrinsic mitochondrial pathway in a ceramide-dependent manner [600]. Taken together, CB2R is the prominent receptor subtype that might play a crucial role in tumor treatment with no psychotic effect.

The molecular mechanisms underlying the antitumor effects of cannabinoids in the CNS are not well understood. Future studies are warranted to determine whether these effects are direct or indirect. In addition to Δ^9^-THC, the antitumor role of CBD is well known and works through multiple signaling pathways, including P38, PPARγ, TRPV2, mTOR as well as COX-2 [524,601]. How CBD served as an effective therapeutic option in different types of brain neoplasia is largely elusive.

Glioblastoma (GBM) is one of the deadliest and most aggressive types of primary brain tumor. It is believed that half of the newly diagnosed gliomas represent GBM. In addition to primary glioblastoma that develop de novo, secondary GBM emerges from glioma of a lower grade [602,603]. Previous studies have shown relatively high expression of CB2R in GBM. Furthermore, increased levels of eCBS and AEA, as well as reduced activity of enzymes involved in the synthesis and degradation of eCBS, including NAPE-PLD and FAAH, have been reported in GBM [604]. Growing evidence support Δ^9^-THC and CBD-mediated suppression of cell proliferation and cell death promotion in vitro using GMB cultured cell through activation of different signaling pathways. Previous studies reported that use CBD in combination with Δ^9^-THC, resulting in an enhanced inhibitory effect of GBM cell proliferation and survival by modulation of ERK1/2 and caspase activities in addition to apoptosis and formation of ROS [605,606,607]. Clinical trials support the use of Δ^9^-THC in the treatment of recurrent GBM [608,609]. Several previous studies support the anti-tumor effect of cannabinoids either through in vivo administration of Δ^9^-THC in tumor patients or in vitro using different brain tumor cultured cell model. Cannabinoid-mediated inhibition of tumor cell proliferation is associated with the induction of apoptosis, modulation of multiple signaling pathways, blocking of angiogenesis, and inhibition of migration and invasion. These observations suggest that cannabinoids not only elicit direct effects but also exert cell cycle arrest in GBM cells upon treatment with Δ^9^-THC and CBD. Scott et al., described inhibitors of heat shock protein in combination with CBD, showing enhanced anti-tumor effects in glioma and GBM treatment [610]. Furthermore, enhanced levels of heat shock protein showed a loss in cytotoxic effect of CBD in glioma cells [610]. In conclusion, endocannabinoids with potential anti-tumor ability along with a significant role in palliative care might serve as an effective therapeutic intervention in improving quality of brain cancer patients.

## 32. Beneficial Effects of Cannabis at the Cost of Risk

The use of cannabis has been highly controversial in view of the physicians, the judicial system, law maker and society. These different views often contradict whether cannabis is beneficial or harmful. The use of cannabis for medicinal purposes is gaining popularity at the cost of serious but poorly understood and limited knowledge of associated risk factors. These risk factors include morphological, neurochemical, behavioral and, importantly, cannabis use disorder in adults. Previous studies have shown that cannabis use does not play any role in improving pain or any other behavioral abnormalities, including depression and anxiety rather user are at high risk of cannabis use disorder [611]. The use of cannabis during pregnancy with the concept that cannabis is harmless is a major concern to our healthcare system and society. The maternal use of cannabis during pregnancy and at the time of breast feeding is not only harmful for mother but exposes fetus to serious problem pre-and postnatally, at the stage of adolescent and adulthood as well. The intensity of damaging effects of cannabis depends on age, mode of application, and the amount and frequency of cannabis use. Growing evidence supports cannabis use as early as at the age of 14 years for recreational purposes. This early use of cannabis leads to multiple developmental changes including neurological abnormalities with high incidences of addiction with prolonged use of Δ^9^-THC. Gilman et al., reported that people with medical marijuana cards are at high risk to cannabis use disorders without any benefit in pain, depression and anxiety. the condition for medical marijuana card. Also, these individuals are reported to be at high risk of developing an addiction. It is of great interest to determine whether the consequences of cannabis used for recreational purposes or used for medicinal needs, specifically by medical marijuana card holders, are the same or different.

## 33. Cannabinoid Receptors Cross-Talk with Other Cell Surface Receptors: A New Way for Cannabinoids Improved Functionality and Diversity

The unwanted psychotic side effects associated with the use of Δ^9^-THC and other related drugs pose certain limitation in the therapeutic uses of cannabinoids. In CNS, dopamine-rich brain regions including ventral tegmental area and hippocampus [5,612] and the incidence of side effects with Δ^9^-THC is an indication of possible functional communication between DR and CBR subtypes. Busquets-Garcia et al., described the role of CB1R present in adrenergic and nonadrenergic cells in stress-induced impaired object recognition memory [613]. Whether, the interaction between adrenergic and CB1R play any role in impaired memory was not well defined but has been predicted [613]. It is possible to predict that the clinical benefits of cannabinoids can be boosted with the help of protein–protein interactions and developing chimeric molecules with increased efficacy and with minimum threat of psychosis and other related side effects. Pain modulation, analgesic effect, anti-inflammation, and neuroprotection are amongst the prominent clinical role of cannabinoids in addition to palliative care in cancer patients. A similar effective role is also seen with many other members of the GPCR family, which are structurally similar, expressed in the same cells and exert comparable physiological and pharmacological effects to the same extent as seen by cannabinoids. These observations are critical and raise the question of whether these receptor proteins functionally interact and are reciprocal in the modulation of certain biological effects. Cannabinoids afford neuroprotection in many neuropathological conditions, as discussed above, through activation of two receptors, CB1R and CB2R, and in heteromeric complex with many other GPCRs as well as with different types of orphan receptors. At present, some synthetics and/or natural cannabinoids are in clinical use in MS patients. However, still, there is hesitation to use cannabinoids in neurodegenerative diseases due to the fear of unwanted side effects and, importantly, death was recently seen with the use of FAAH inhibitors [614]. To minimize the risk factor with the use of cannabinoids with improved physiological significance and therapeutic values of cannabinoids, targeting receptors in heteromeric complex with chimeric molecules might serve as an effective therapeutic avenue. This combination holds some significance and promises to minimize the risk of untoward effects of cannabinoids as predicted with the interaction between CB1 and CB2 receptors and with other receptors [121,415,615,616,617,618]. The concept of CBR dimerization stems out from previous studies describing modulation of excitatory and inhibitory neurotransmitters, increased release of glutamate in response to CB2R activation via involving ERK signal transduction pathway, inhibition of GABA release in response to CB1R activation, and its effect on synaptic currents provide compelling evidence in support of interactions between CBR subtypes with glutamate and GABA receptors.

The dose-dependent effect of Δ^9^-THC and increased risk of psychosis are well established, and with the psychotic effect of Δ^9^-THC, most studies are directed towards the DAergic system with the effect of cannabinoids. Although, the presence of CBR subtypes on multiple neurons in different brain regions expressing multiple neurotransmitters, including GABAergic and glutamatergic neurons, has been documented, the functional consequences of such association are not well established. CB1 receptors, upon activation, not only resulted in inhibition of neuronal activity but also modulated the release of some key neurotransmitters, including serotonin, GABA, acetylcholine, DA, histamine, glutamate and noradrenaline, and help in maintaining the balance between excitatory and inhibitory input in CNS.

In the CNS, the expression level of CB1R is relatively higher in GABAergic than glutamatergic neurons [619]. Furthermore, decreased GABA release has been reported upon activation of neurons expressing CB1 receptors on the GABAergic [15,620] and is involved in regulating GABAergic synaptic transmission [317,621,622]. These observations are further strengthened by studies describing the increased GABA signaling of GABAergic neurons in the absence of CB1R on them. Furthermore, studies have shown gradual neuronal loss in hippocampus and inflammation in mice displaying GABAergic neurons devoid of CB1R expression. Taking these results together, it was proposed that ECS serve as a communication block between glial cells and neurons, which might be true in the hippocampus for GABAergic neurons and microglial cells because microglial cells produce more EC than neurons with high expression of CB1R in GABAergic neurons [619]. Colizzi et al., described that Δ^9^-THC affects glutamate signals in humans [623]. Previous studies have also demonstrated suppressed levels of glutamate-associated metabolites in the basal ganglia upon chronic use of cannabis [624]. Whereas with the use of cannabis, gender-related changes in glutamate metabolites have also been reported specifically in the dorsal striatum [625]. However, in brain regions including the cingulate cortex, such changes are controversial with the use of cannabis [626,627,628]. While CB2R is devoid of psychoactivity, unlike CB1R, CB1R and CB2R exert overlapping functions, including cell proliferation and a cell survival neuroprotective role in neurodegenerative diseases. Furthermore, studies have also demonstrated colocalization of CBR subtypes in different brain regions, suggesting possible cross-talk between CBR subtypes. In this section of the present review, the physiological relevance and therapeutic advantages are discussed with respect to homo-and heterodimerization of CBR subtypes with some other key cell surface receptor proteins of the GPCR family. In accordance with the overlapping function and regulatory mechanism here, homo-and heterodimerization of CBR within the family and with other GPCRs is discussed.

### 33.1. Homo-And Heterodimerization of CB1R and CB2R

By using multiple techniques, including morphological (colocalization), biochemical (Co-immunoprecipitation), and biophysical, including fluorescent resonance energy transfer (FRET) analysis and bioluminescence resonance energy transfer (BRET) the preexisting concept that GPCR exists as monomers have been challenged. Growing evidence supports that CBR subtypes exhibit homo-and heterodimerization within the family and with other cell surface receptors. Earlier observations also support that GPCRs exist in higher-order oligomers. CB1R is a prominent receptor in CNS, whereas CB2R primarily was believed to be expressed in peripheral tissues but also present in CNS. Both CB1R and CB2R are a member of the GPCR family and couple to Gi/Go and are involved in different array of functions. Like many other Gi-coupled GPCRs, CB1R and CB2R following activation in the presence of agonists lead to the inhibition of AC and formation of cAMP as well as PKA as discussed above. Previous studies using specific antibodies which recognize CB1R homodimer have shown that CB1R exists as a homodimer and even higher order of oligomers [102,629,630,631]. Furthermore, CB1R dimerization is regulated in an agonist-dependent manner. Whether CB2R exist and function as a monomer and dimer is not well understood.

Indeed, the co-activation of CB1R/CB2R heterodimers in SH-SY5Y cells abolishes neuritogenesis induced by either receptor agonist [615]. These results suggest that the activation of two receptors at a time might mask the effect of one receptor. The formation of a functional heteromeric complex between CB2 and CB1 receptors in transfected neuronal cells and in rat brain pineal gland, nucleus accumbens, and globus pallidus has been described [615]. Furthermore, simultaneous activation of CB1 and CB2 receptors in a complex elicits a negative effect on the status of Akt phosphorylation and neurite outgrowth. In such a heteromeric complex, antagonists to either CB1R or CB2R prevent each other effects and support bidirectional cross-antagonism [615]. Microglial cells are rich with CBR subtypes, specifically CB2R, and during neurodegeneration, activated microglial cells displayed increased heteromeric complex formation between CB1 and CB2R that work on proinflammatory molecules and resulted in suppression of inflammation [616]. Although, both CB1R and CB2R are well expressed in glial cells, especially in astrocytes and microglial cells and play a neuroprotective role in inflammation-associated neurological diseases, the pathological significance and molecular mechanism are still elusive. Since, CB2R highly expressed in glial cells, the activation of this receptor needs to be explored in inflammation-associated neurological disease such as MS. Whether such advantages of CBRs heterodimerization also exist in other neuropathological condition is not known. However, the complex formation between CB1R and CB2R has been reported in basal ganglia primarily at the postsynaptic site. Furthermore, heterodimerization between CBR subtypes has also been described in the PD model of primate that was reduced in animal displaying dyskinesia in response to levodopa [617]. It will be interesting to explore such an avenue in a condition where receptor expression and the levels of endocannabinoids are changed in a receptor-specific manner.

### 33.2. Heterodimerization of Cannabinoid Receptor with Other GPCRs

In the last 20 years, several seminal observations have changed the pre-existing concept of GPCRs pharmacology through the formation of homo-and heterodimers. The formation of homodimers and heterodimers at cell surface or intracellularly represent new receptors with a different function from its native receptors. Co-expression of CBRs with other GPCRs at the cell surface or intracellularly and regulation of multiple neurotransmitters release is the possible indication that CBRs might work in concert as homo and heterodimers. GPCRs, when co-expressed in a single cell, often display physical and functional interaction. The functional interaction in a heteromeric complex between CBRs is not only limited within the family; it has also been described with other GPCRS. There is growing evidence that CB1R exhibits heterodimerization with CB2R, Dopamine, adenosine, angiotensin, orexin, opioid and somatostatin receptors [615,632,633,634,635,636,637,638,639].

### 33.3. CBR and Opioid Receptors Heteromeric Interaction and Consequences

Opioid receptors (OPRs) have shown great diversity in response to ligands when coexpressed within the family and members of other GPCRs, including CBR subtypes. Multiple evidence at the anatomical, physiological and pharmacological level supports the cross-talk between OPR and CBR subtypes. Both receptors belong to the GPCR family, display structural and sequence similarity, are widely distributed in most brain regions, including spinal cord and via coupling to Gi inhibit AC and involved in negative regulation of Ca^2+^ channel, influx and regulation of many neurotransmitters following receptor activation [529]. Most importantly, OPR and CBR colocalized in GABAergic neurons [640]. Furthermore, there is evidence supporting ligand-dependent regulation of such cross-talk, indicating the possibility of heterodimerization. The heterodimerization between CBR and μOPR using BRET analysis has been described [641]. The functional significance and changes in receptor pharmacology following heterodimerization are also well supported [642,643]. In addition, the changes in MAP kinases have also been documented following ligand-dependent activation of CBR and OPR [641]. Canals and Milligan demonstrated that CB1R with coexpression with µOPR suppressed µOPR function [147]. In addition to cross-talk between CBR and OPR subtypes, biochemical and behavioral studies also support cross-tolerance between these two prominent members of the GPCR family.

### 33.4. Heterodimerization Between CBR and Dopamine Receptors

CB1R’s first heterodimerization was reported with D2R which showed that CB1R and D2R exist in a constitutive heteromeric complex, that is strengthened in the presence of CB1R agonists, whereas decreases in the presence of inverse agonist. In the case of CB1R and D2R, it is worth mentioning the functional consequences of this complex formation. Recent observations revealed antiparkinsonian effect of CB1R antagonist in heteromeric complex of CB1R and D2R [644]. Kearn et al., using immunoprecipitation and Western blot analysis, showed constitutive heterodimerization between CB1R and D2R, which was strengthened in the presence of CB1R agonist whereas decreased with inverse agonists [645]. Moreover, D2R is endogenously expressed in STHdhQ7/7 cells and forms a heterodimer with CB1R [646]. Co-activation of D2R influences CB1R coupling to G protein and β-arrestin-mediated ERK1/2 signaling [183,645,646,647]. In addition, the existence of antagonistic CB1R and D2R interactions within CB1R/D2R heterodimers has also been reported, and this study further emphasized the possibility of interaction with A_2A_ receptor [648].

### 33.5. Heterodimerization Between CBR and Somatostatin Receptors

SSTRs and CB1R are co-expressed in different brain regions in a receptor-specific manner [618]. Our results showed that CB1R and SSTR5 colocalized in the rat brain cortex, striatum, and hippocampus. CB1R was expressed in SSTR5 immunoprecipitate prepared from the brain tissue lysate, indicating their association in a system where these receptors are endogenously expressed. We recently described heterodimerization between SSTR2 and SSTR5 with CB1R in mutated HD striatal cultured neurons [121,122,415,618]. In cotransfected HEK-293 cells, SSTR5 and CB1R exist in a constitutive heteromeric complex under basal conditions, which was disrupted upon agonist treatments. Furthermore, the concurrent activation of SSTR5 and CB1R led to the preferential formation of SSTR5 homodimer and dissociation of CB1R homodimer. In conclusion, our study suggests that in a recombinant system, CB1R forms heterodimer with SSTR5, which leads to SSTR5-mediated dominant signaling in the cAMP/protein kinase A/ERK pathway [618]. We further extended our study and determined whether interaction exists in a pathological condition. CB1R expression was determined in SSTR2 or SSTR5 immunoprecipitation using Co-IP in STHdhQ7/7 and STHdhQ111/111 cells following treatment with receptor-specific agonists alone and in combination. Immunoprecipitate prepared was processed for receptor complex formation between SSTR2/CB1R and SSTR5/CB1R, respectively. Our results showed receptor and agonist-specific changes between CBR and SSTR subtypes in control and cells with extended CAG repeats with a significant impact on the regulation of signaling pathways [618].

Furthermore, the interaction between CB1R and b2-adrenoceptor has also been reported in HEK-293 as well as in HTM cells [649]. Rozenfeld et al., described that the formation of AT1R–CB1R heteromeric complex showed changes in signaling in response to angiotensin II in stellate cells prepared from ethanol-treated cells when compared to control. Authors also claimed that such an effect was encountered following the blockade of CB1R activity [637]. Previous studies have also shown that JWH-015, which binds to CB2R, mediated modulation of breast cancer growth and invasion in combination with chemokine receptor type 4 [650]. It is interesting to note that CB2R also interacts in a heterodimeric complex with orphan GPCR, namely GPR55, and modulates cancer cell signaling as potential therapeutic interference of Δ^9^-THC (with appropriate dose) in cancer [470]. The expression of CBR has been reported in GABAergic interneurons in CNS. Like CBRs, the therapeutic beneficial effect GABA_B_ receptor in pain, epilepsy and psychiatric disorders in addition to co-expression in different brain regions indicating cross-talk between CB1R and GABA_B_ receptor but has not been elaborated in detail yet. Studies also support CB1R and GABA_B_ receptor action in a reciprocal manner as well as noted contribution in the regulation of cognitive function in the hippocampus and antagonists for both receptors in different animal models improve cognitive function. Cinar et al., using binding studies, described interaction between these two receptors and showed inhibition of GABA_B_ -mediated effect on G protein signaling in the presence of CB1R antagonist AM251 [651]. In addition to these observations, previous studies have also described the existence of GPR55 and CB2R in some pathological conditions, including human tumors [470,652]. The endogenous cannabinoid PEA, which works in CBR independent manner, served as an agonist to GPR55 and resulted in augmented GABA transmission in striatum along with the synthesis of 2-AG that inhibits GABA release in a retrograde manner [653]. Furthermore, PEA-mediated regulation of GABA is completely blocked following inactivation of GPR55 and can be mimicked in the presence of GPR55 synthetic agonist and supporting the possible cross-talk between GABAergic neurotransmission and endogenous cannabinoids [653]. In this direction, the synthesis of new drugs targeting CB2R in a complex need to be explored in neurological diseases. Furthermore, future studies addressing the role of CBR subtypes, specifically CB2R cross-talk with other GPCRs implicating in the regulation of synthesis of endocannabinoids or inflammatory and signaling pathways in neurological diseases, will delineate molecular mechanisms for the role of CBR subtypes.

## 34. Perspective, Future Direction, and Conclusions

The discovery of the ECS has sparked the interest of many researchers worldwide due to its potential therapeutic contribution to some of the incurable neurodegenerative diseases such as AD, PD, HD, and psychological abnormalities. To date, studies have uncovered the expression, location, structures, and mechanism of cannabinoid receptors. When the endocannabinoid system’s associations with other biochemical pathways are fully elucidated, many medical and political changes will be seen, such as the legalization of marijuana and new therapeutic approaches to neurodegenerative diseases. Recent developments regarding crystal structure and cryoEM open the door to understanding the structural complexity and future therapeutic implication of cannabinoids in neurological and psychiatric disorders. Most genes associated with neurological diseases have been defined; however, the molecular details of other changes are largely elusive and are of immense interest to be explored. At this stage, it will be interesting to elucidate the role of CB2R as a neuroprotective strategy in addition to other proteins that are modulated following cannabis administration. Neuroinflammation, oxidative stress, and disrupted cell organelles, specifically mitochondria, are intimately associated with compelling causative factors for disease progression and are potential therapeutic avenues to explore in neurodegeneration, along with psychological disturbances; therefore, they should be the prime objective for future studies on cannabinoids to develop novel therapeutic chimeric molecules with minimum side effects and maximum benefits.

## Abbreviations

AD Alzheimer’s disease, CB1R cannabinoid CB1 receptor, CB2R cannabinoid CB2 receptor, CBD cannabidiol, CNS central nervous system, FAAH fatty acid amide hydrolase, GPCR G-protein-coupled receptor, PD Parkinson’s disease, PET positron emission tomography, PPARγ peroxisome proliferator-activated receptor γ, 2-AG, 2-arachidonoylglycerol; AA, arachidonic acid; ACEA, arachidonyl-2′-chloroethylamide; AEA, anandamide; BDNF, brain-derived neurotrophic factor; CB, cannabinoid receptor; CBC, cannabichromene; CBD, cannabidiol; CREB, cAMP response element-binding protein; DAGL, DAG lipase; FAAH, fatty acid amide hydrolase; GFAP, glial fibrillary acidic protein; IGF-1, insulin-like growth factor-1; mTORC1, mammalian target of rapamycin complex 1; NGF, nerve-growth factor; NPC, neural progenitor cell; NSC, neural stem cell; SGZ, subgranular zone; Shh, Sonic Hedgehog; Smo, smoothened; SVZ, subventricular zone; THC, Δ^9^-tetrahydrocannabinol; TRPV1, transient receptor potential cation channel subfamily V member 1.
